# Comparative Genomics of *Chloropicon primus* and *Chloropicon roscoffensis* Provide Insights into the Evolutionary Dynamics and Ecological Success of These Tiny Green Algae in Marine Environments

**DOI:** 10.1093/gbe/evaf140

**Published:** 2025-07-11

**Authors:** Monique Turmel, Jean-François Pombert, Christina Kosanovic, Alexander Thomas Julian, Christian Otis, Claude Lemieux

**Affiliations:** Département de Biochimie, de Microbiologie et de Bio-Informatique, Institut de Biologie Intégrative et des Systèmes, Université Laval, Québec, QC G1V 0A6, Canada; Department of Biology, Illinois Institute of Technology, Chicago, IL, USA; Department of Biology, Illinois Institute of Technology, Chicago, IL, USA; Department of Biology, Illinois Institute of Technology, Chicago, IL, USA; Département de Biochimie, de Microbiologie et de Bio-Informatique, Institut de Biologie Intégrative et des Systèmes, Université Laval, Québec, QC G1V 0A6, Canada; Département de Biochimie, de Microbiologie et de Bio-Informatique, Institut de Biologie Intégrative et des Systèmes, Université Laval, Québec, QC G1V 0A6, Canada

**Keywords:** Chloropicophyceae, ploidy change, CO_2_ concentrating mechanism, Calvin–Benson cycle, pentose phosphate pathway, queuosine metabolism

## Abstract

The tiny green algae belonging to the class Chloropicophyceae play a key role in marine phytoplankton communities, especially in moderately oligotrophic water; yet, little is known about their biology, lifestyles, and what allows them to thrive in various oceanic environments. A single representative of this class (*Chloropicon primus*), comprising eight recognized species, has been previously subjected to genome analysis. To gain insight into the evolutionary changes that occurred during speciation in the *Chloropicon* genus and better understand the genes that distinguish *Chloropicon* species from other green algae traditionally designated as prasinophytes, we sequenced the genome of a second strain of *C*. *primus* and those of three strains of the closely related *Chloropicon roscoffensis*, the latter species representing the most dominant *Chloropicon* lineage in oceans. Our analyses highlighted substantial interspecific variations, including differences in chromosome number, gene content, gene arrangement, and ploidy state. Both *C*. *primus* genomes were predominantly diploid, while the *C*. *roscoffensis* genomes were either haploid or diploid. Specific proteins were identified for each species. *Chloropicon roscoffensis* possesses a biochemical C_4_-like inorganic carbon concentrating mechanism that potentially enables recycling of mitochondrial CO_2_ derived from photorespiration and respiration for carbon fixation in the chloroplast. In addition, it features specific proteins linked to the central carbon metabolism that suggest better coping mechanisms for abiotic stresses compared to *C. primus*. We also uncovered a previously undescribed eukaryotic recycling pathway for the micronutrient queuosine, a hypermodified nucleoside that is essential for post-transcriptional modification of several tRNAs at their anticodon wobble position.

Significance It remains largely unknown what makes the tiny green algae belonging to the Chloropicophyceae successful in marine waters, especially in environments poor in nutrients, as only the genome of a single species (*Chloropicon primus*) has been decoded so far. To address this question and also to understand why the closely related *Chloropicon roscoffensis* is more dominant than *C. primus* in the oceans, we set out to identify the genomic changes that occurred during the divergence of these species using a comparative genomics approach. Substantial genomic variations were found between *C. primus* and *C. roscoffensis*, including differences in chromosome number, ploidy state, gene content, and metabolic pathways. These genomic variations likely play an important role in the adaptability of *Chloropicon* species to different oceanic environments.

## Introduction

The unicellular green algae traditionally designated as prasinophytes constitute a heterogeneous assemblage of photosynthetic organisms, with the great majority representing paraphyletic lineages at the base of the Chlorophyta, the sister lineage of the Streptophyta (Leliaert et al. 2012; Bachy et al. 2022). A third phylum of the Viridiplantae comprising the Prasinodermophyta has recently been recognized (Li et al. 2020a), but its sister position to the Chlorophyta and Streptophyta remains controversial (Yang et al. 2023). Given their phylogenetic positions, prasinophytes hold the key to understanding the nature of the last common ancestor of all green plants and the origin of core chlorophytes. They exhibit considerable diversity with respect to cell shape and size, flagella number and behavior, mitotic and cytokinetic mechanisms, and biochemical features such as accessory pigments and storage products (Leliaert et al. 2011). Early analyses of the nuclear-encoded small subunit (18S) rRNA gene identified nine monophyletic groups of prasinophytes (clades I through IX), with clades VIII and IX composed uniquely of environmental sequences (Leliaert et al. 2011). Most prasinophyte clades have been ranked at the level of classes, with the most recently erected one being the Pseudoscourfieldiophyceae (Chlorophyta) (Crépeault et al. 2024).

Prasinophytes abound in oceans and contribute significantly to carbon dioxide capture and sequestration through photosynthesis. Yet, despite their ecological importance, very little is known about their biology, lifestyles, and what allows them to thrive in different environments of the oceans. To unravel their evolutionary trajectories, sequencing approaches targeting their genomes or transcriptomes have been privileged. So far, high-quality genomes have been reported for several species of the Mamiellophyceae (Derelle et al. 2006; Palenik et al. 2007; Worden et al. 2009; Moreau et al. 2012; van Baren et al. 2016), one species of the Chloropicophyceae (Lemieux et al. 2019), and one member of the Prasinodermophyta (Li et al. 2020a). Based on sequence data and cytometry, the estimated genome sizes of the Mamiellophyceae examined so far vary between 12.6 (*Ostreococcus tauri*) and 303 (*Monomastix opisthostigma*) Mb, with three of the five Mamiellales genera (*Ostreococcus*, *Bathycoccus*, and *Micromonas*) displaying genomes smaller than 22 Mb (Yung et al. 2022). Species from the latter genera are haploid and display 17 to 21 chromosomes (∼8,000 to 11,000 genes), two of which have an atypically low GC content (Piganeau et al. 2011; Grimsley et al. 2015). Within each of these three genera, dynamic genome evolution was linked to species divergence or adaptation to ecological niche (Grimsley et al. 2015).

The Chloropicophyceae are widely distributed in oceans and play a key role in marine phytoplankton communities, especially in moderately oligotrophic water (Lopes dos Santos et al. 2017a; Lopes dos Santos et al. 2017b). These picoalgae are lacking flagella, scales, and a pyrenoid, and compared with the Mamiellophyceae, they are positioned closer to the common ancestor of all core chlorophytes in the tree of life (Lemieux et al. 2014; Leliaert et al. 2016; Lopes dos Santos et al. 2017b; Turmel et al. 2019). Based on phylogenies inferred from the 18S rRNA gene (Lopes dos Santos et al. 2017b), later confirmed by a chloroplast phylogenomic study (Turmel et al. 2019), it was found that the Chloropicophyceae comprise at least eight distinct species that form two clades, designated as the *Chloropicon* and the *Chloroparvula* clades. Like its Mamiellales counterparts, the nuclear genome of *Chloropicon primus* CCMP1205, originally collected from the Atlantic Ocean, is among the smallest known in the Viridiplantae (Lemieux et al. 2019). With a haploid genome size of 17.4 Mb, it encodes 8,693 genes and consists of 20 chromosomes, all of which are present in two copies, except for chromosome 17, which is present in three copies. The *C. primus* genome is one of the first diploid genomes documented in the Chlorophyta. Many genes encoding flagellar proteins were identified in this genome, implying that the life cycle of chloropicophyceans may include a flagellar stage that has not been observed yet (Lemieux et al. 2019). The absence of a large set of genes that are found in the Mamiellophyceae and in most or all other previously investigated green algal lineages suggests that genome minimization occurred separately in the Chloropicophyceae and Mamiellophyceae. Among the genes identified in *C*. *primus* but not in the Mamiellales are those encoding a complete set of enzymes performing the degradation of propionyl-CoA via the 2-methylcitrate cycle, as well as proteins required for the synthesis of astaxanthin and thiamine, and enzymes participating in degradation of branched-chain amino acids (Lemieux et al. 2019).

The present study was undertaken with two main goals: (i) gain insight into the changes that the nuclear genome underwent during evolution in the *Chloropicon* clade and (ii) improve our knowledge about the genes that distinguish *Chloropicon* species from other prasinophytes and chlorophytes. To reach these goals, we sequenced the genome from a second isolate of *C. primus* (RCC138) as well as the genomes from three isolates of *Chloropicon roscoffensis* that were collected from the Pacific Ocean (RCC2335) and different areas of the Atlantic Ocean (CCMP1998 and RCC1871). It is worth mentioning here that *C*. *primus* tends to be found in oligotrophic environments, whereas *C*. *roscoffensis* occurs more often in coastal environments (Lopes dos Santos et al. 2017a), implying that the eventual discovery of gene content differences between these two species might be linked with variations in nutrients or other environmental factors. In fact, our comparative genome analyses uncovered multiple differences in chromosome architecture and gene content between *C*. *primus* and *C*. *roscoffensis,* with the isolates from the latter species exhibiting variations in ploidy level and numerous extra genes relative to *C. primus.*

## Results

### Architecture and Ploidy of *Chloropicon* Genomes

The *C. roscoffensis* (strains RCC2335, RCC1871, CCMP1998) and *C. primus* (strain RCC138) genomes were sequenced and assembled from telomere-to-telomere into 19 and 20 chromosomes totaling 16.8 and 17.5 Mb, respectively (Table 1), with telomere repeats missing only from one end of chromosome 1 in *C. primus* RCC138 and from a few select contigs in *C. roscoffensis* RCC1871 (Table S1). The telomeric repeat units capping the chromosomes were found to be identical between the *Chloropicon* species and ranged in size from the TTTAGG hexamer to the TTTTTAGG octamer in all genomes. The *Chloropicon* genomes are very compact, with a high gene density and <1 intron per gene on average, as well as a low proportion of repeated sequences (Table 1).

**Table 1:** General characteristics of the *Chloropicon* nuclear genomes analyzed in this study

	*Chloropicon primus*		*Chloropicon roscoffensis*		
	RCC138	CCMP1205	RCC2335	CCMP1998	RCC1871
Clade	VII A2	VII A2	VII A4	VII A4	VII A4
Sampling information					
Ocean	Pacific	Atlantic	Pacific	Atlantic	Atlantic
Location	North Pacific	Gulf Stream	Sagami Bay	Sargasso Sea	English Channel
Isolation date	1992	1965	2009	1998	2009
Assembly					
Haploid genome size (bp)	17,584,162	17,400,691	16,791,247	16,832,509	16,820,234
G + C content (%)	57.44	57.52	60.43	60.42	60.5
Repeats (%)	9.09	8.29	5.50	5.98	5.97
Chromosomes (no.)	20	20	19	19	19
Longest chromosome (bp)	1,855,637	1,876,603	2,040,242	2,046,996	1,787,833
Shortest chromosome (bp)	417,210	368,919	326,295	327,266	367,329
Telomere repeat	(TT)TTTAGG	(TT)TTTAGG	(TT)TTTAGG	(TT)TTTAGG	(TT)TTTAGG
Ploidy state	mostly diploid	mostly diploid	haploid	haploid	diploid
Heterozygous sites (no.)	3,441	7,714	302	642	46,222
Heterozygous sites (no./kb)	0.20	0.44	0.02	0.04	2.75
Genes					
Predicted genes (no.)	8,683	8,693	8,974	8,975	8,976
Protein-coding genes (no.)	8,627	8,639	8,922	8,925	8,923
Ribosomal RNA genes (no.)	10	8	11	11	11
Transfer RNA genes (no.)	46	46	41	39	42
Gene density (gene/kb)	0.494	0.5	0.534	0.533	0.534
Genes with introns (%)	37.44	36.32	43.82	44.20	43.81
Introns (average no./gene)	0.54	0.52	0.72	0.73	0.73
Average exon size (bp)	975	971	840	847	840
Average intron size (bp)	152	154	122	122	122

All values reported in this Table are associated with the representative haploid genomes.

**Fig. 1.**
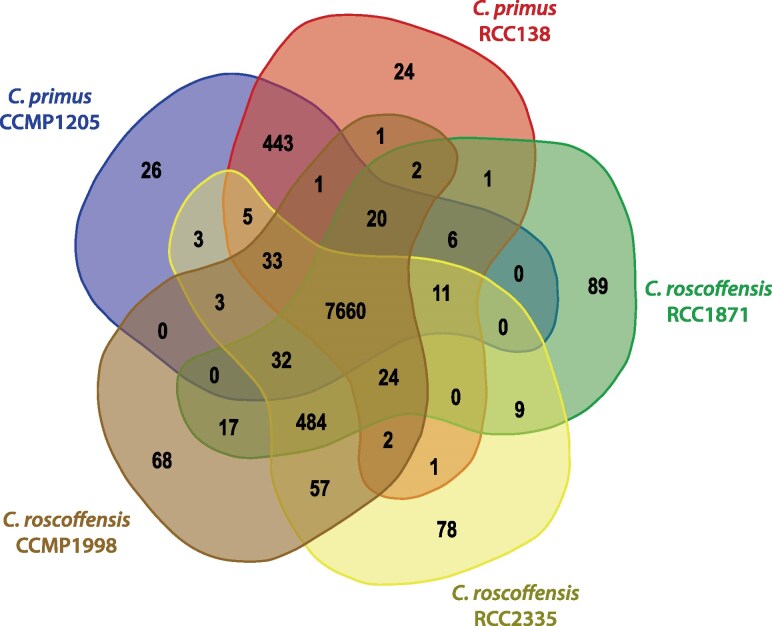
Venn diagram of protein orthogroups shared by or unique to the *Chloropicon primus* and *Chloropicon roscoffensis* strains analyzed in this study.

The predicted proteins across all *Chloropicon* genomes were grouped into 9,100 distinct orthogroups (Fig. 1). Of these, 7,660 orthogroups (84.2%) were found to be shared among all isolates. A total of 802 orthogroups—comprising 851, 913, and 827 genes in CCMP1998, RCC1871, and RCC2335, respectively—were unique to *C. roscoffensis*, while 493 orthogroups—containing 645 and 647 genes in CCMP1205 and RCC138, respectively—were exclusive to *C. primus* (Fig. 1; Files S1 and S2). These species-specific orthogroups account for 9.2% to 10.2% of the genes in *C. roscoffensis* and 7.4% to 7.5% of those in *C. primus*. It should be noted here that the identification of species-specific orthogroups by OrthoFinder is sensitive to the inflation parameter (option -I); using a value higher than the default might have resulted in the detection of fewer orthogroups. Analysis of gene family evolution across the 9,100 orthogroups revealed that only a small fraction (4.2%) experienced expansions or contractions (Figs. S1 and S3). Notably, gene family expansions were more prevalent in the *C. roscoffensis* lineages than in those of *C. primus*.

**Fig. 2.**
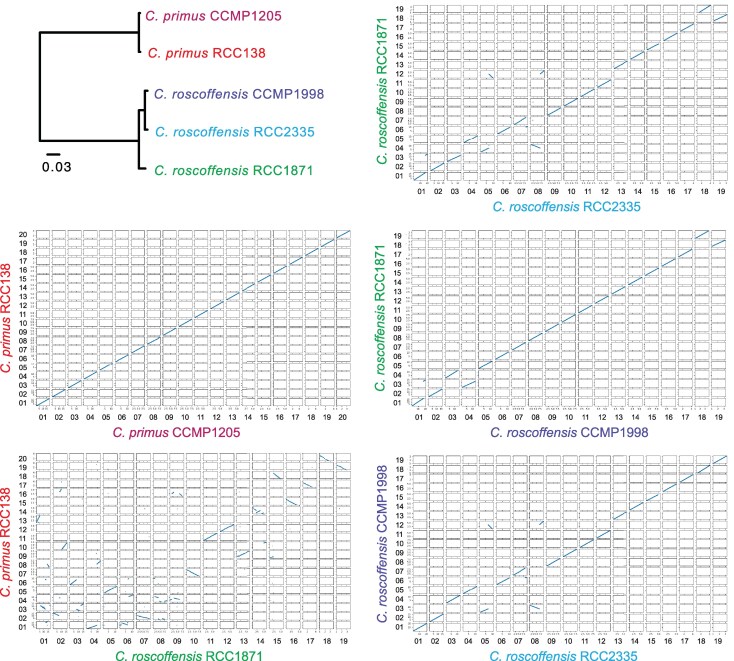
Phylogenetic tree showing the relationships among the five examined *Chloropicon* strains and dot plots comparing the chromosomes of selected genome pairs. The tree was inferred from the 7,281 single-copy orthologous proteins shared by these strains.

Phylogenetic analysis of the proteins shared by all isolates showed that *C. roscoffensis* strains CCMP1998 and RCC2335 form a strongly supported clade, with *C. roscoffensis* RCC1871 occupying a sister position relative to this clade (Fig. 2). Values of average nucleotide sequence identity (ANI) determined using FastANI were in agreement with the protein phylogenomic tree: the ANI for each genome pair is 77.88% (*C*. *primus* RCC138 and *C*. *roscoffensis* RCC1871), 99.91% (*C. primus* CCMP1205 and RCC138), 99.77% (*C*. *roscoffensis* RCC2335 and CCMP1998), 98.25% (*C*. *roscoffensis* RCC1871 and CCMP1998), and 98.25% (*C*. *roscoffensis* RCC1871 and RCC2335).

**Fig. 3.**
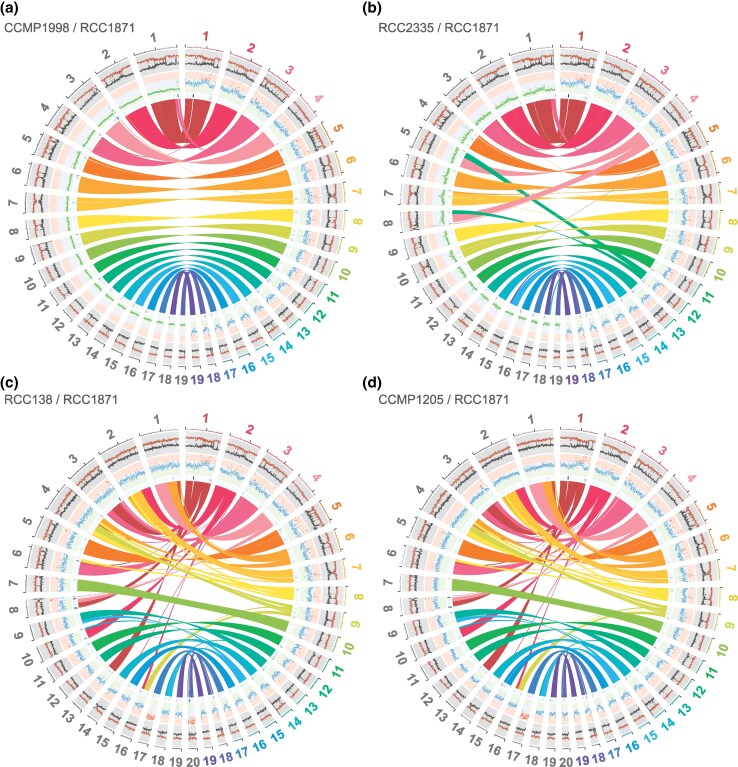
Circos maps comparing the genome of *Chloropicon roscoffensis* RCC1871 with those of other *Chloropicon* species: a) *C. roscoffensis* CCMP1998; b) *C. roscoffensis* RCC2335; c) *C. primus* RCC138; d) *C. primus* CCMP1205. GC and AT percentages are plotted in red and grey, respectively, in the outermost concentric circles (sliding window 10,000 nt, step 5,000 nt). Normalized sequencing depths (Illumina) are plotted in the central concentric circles, with haploid, diploid, and triploid depths being color coded in green, blue, and red, respectively (sliding window 10,000 nt, step 5,000 nt). Densities of repeat elements identified with RepeatMasker are illustrated by blue heatmaps in the innermost concentric circles. Colinear blocks are shown highlighted by ribbons extending from the *C. roscoffensis* RCC1871 chromosomes to those from the genomes being compared.

Although most of the genes in the *C. roscoffensis* RCC1871 and *C. primus* RCC138 genomes are arrayed collinearly, many chromosomes in these genomes are not collinear along their entire length, indicating that large blocks of genes were frequently relocated to different chromosomes or were shifted in position within individual chromosomes during speciation (Figs. 2 and 3). Nine of the 19 *C*. *roscoffensis* chromosomes show such rearrangements. In contrast, no rearranged gene blocks were observed in the comparison of the two *C. primus* genomes, all chromosomes being entirely collinear with their homologs in the other isolate (Fig. 2; Fig. S2). The comparisons of the three *C*. *roscoffensis* genomes, however, revealed that a few chromosomes were subjected to rearrangements: the genomes of the two strains collected from the Atlantic Ocean, RCC1871 and CCMP1998, only differed by a single fragmentation/fusion event, while those of the RCC1871 strain and the isolate from the Pacific Ocean (RCC2335) featured three single fragmentation/fusion events and two reciprocal translocations (Figs. 2 and 3; Fig. S2).

It was found that the *C. roscoffensis* genomes are either haploid (RCC2335 and CCMP1998) or diploid (RCC1871) with no aneuploid chromosomes, whereas the *C. primus* genomes are predominantly diploid and featured triploid alleles on chromosomes 17 (RCC138 and CCMP1205) and 20 (RCC138) (Table 1; Fig. 3; Fig. S2). As expected, read mapping/variant calling analyses disclosed only a few putative heterozygous loci in the *C. roscoffensis* strains RCC2335 and CCMP1998, in sharp contrast with the numerous heterozygous loci found in the *C. roscoffensis* strain RCC1871 and the two *C*. *primus* strains (Table 1). In the case of the *C. primus* genomes, analyses of both allelic frequency distributions and sequencing depths corroborated the aneuploid nature of chromosomes 17 and 20 (Fig. S3).

### Differences in Gene Content Between the *C*. *primus* and *C*. *roscoffensis* Genomes

Most of the protein orthogroups specific to *C*. *primus* or *C*. *roscoffensis* consist of hypothetical proteins, and among the species-specific orthogroups that include proteins of known functions/domains, there are large gene families that feature transposases, ankyrin repeat proteins and guanylate cyclases (Files S1 and S2). Each *C. roscoffensis* isolate features its own expanded set of guanylate cyclases, which appears to have arisen by gene duplications (RCC1871, OG0000007; CCMP1998, OG0000014; RCC2335, OG0000018; see File S2). The remaining proteins of known functions that are unique to *C*. *roscoffensis* or *C*. *primus* have diverse metabolic or cellular functions. Below, we describe the functions of proteins unique to *C. roscoffensis* that are involved in inorganic carbon concentration and central carbon metabolism.

#### CO_2_ Concentration Mechanisms

**Fig. 4.**
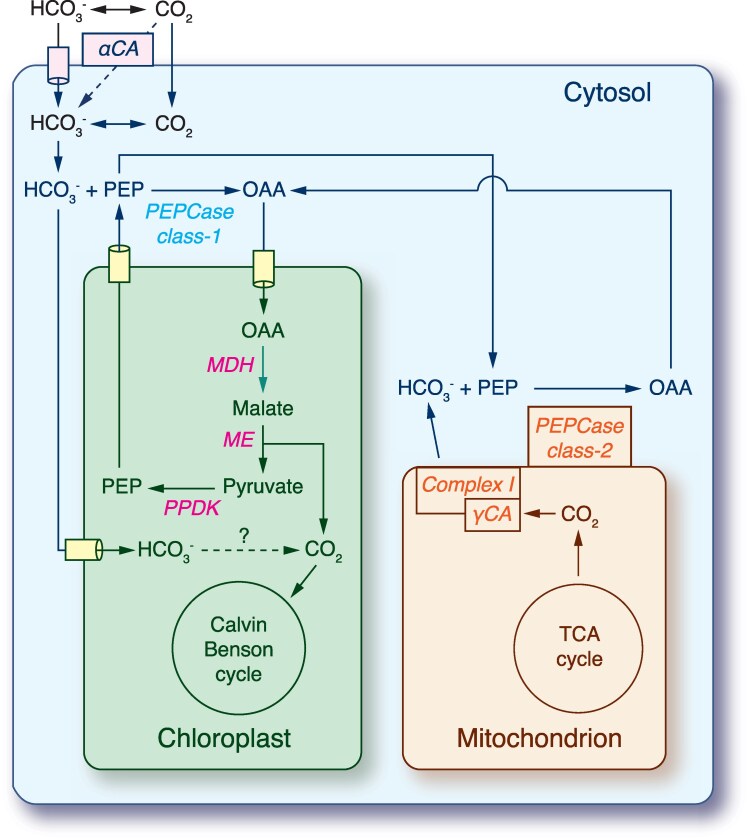
Proposed model for the CCM of *Chloropicon roscoffensis.* αCA, alpha carbonic anhydrase; γCA, gamma carbonic anhydrase; MDH, NADP-dependent malate dehydrogenase; ME, NADP-dependent malic enzyme; PEPCase, phosphoenolpyruvate carboxylase; PPDK, pyruvate, phosphate dikinase.

During evolution, many photosynthetic organisms developed inorganic carbon concentration mechanisms (CCMs) to increase CO_2_ concentration around the ribulose-1,5-bisphosphate carboxylase/oxygenase (Rubisco, EC 4.1.1.39) and thereby reduce photorespiration (Zabaleta et al. 2012). Analysis of the CCM components identified for the *C*. *roscoffensis* isolates (Table 2) suggests that this species can accumulate CO_2_ by dehydrating extracellular bicarbonate (HCO_3_^−^) via a carbonic anhydrase (αCA) and that, in addition, it possesses a biochemical C_4_-like CCM that allows generation of CO_2_ using C_4_ enzymes (Fig. 4). This C_4_-like CCM comprises a carbon pre-fixation step during which HCO_3_^−^ combines with phosphoenolpyruvate (PEP) via PEP carboxylase (PEPCase, EC 4.1.1.31), producing oxaloacetate (OAA) in the cytosol. OAA is subsequently transported to the chloroplast where a NADP-dependent malate dehydrogenase (MDH, EC 1.1.1.82) and a decarboxylating NADP-dependent malic enzyme (ME, EC 1.1.1.40) convert this C_4_ molecule into pyruvate and CO_2_ for final carbon fixation by Rubisco in the Calvin-Benson (CB) cycle. PEP is then regenerated from pyruvate by the pyruvate phosphate dikinase (PPDK, EC 2.7.9.1) and returned to the cytosol for a further round of carboxylation by PEPCase.

**Table 2:** *Chloropicon* proteins involved in inorganic carbon concentration and central carbon metabolism

Protein	Function	Localization	*Chloropicon primus*		*Chloropicon roscoffensis*		
			CCMP1205	RCC138	RCC1871	RCC2335	CCMP1998
Inorganic carbon concentrating mechanism (CCM)							
PEPCase (class-1)	Phosphoenolpyruvate carboxylase	Cytosol	03p27480	03g27480	03g26920	01g11060	01g11210
PEPCase (class-2)	Phosphoenolpyruvate carboxylase	Cytosol	…	…	19g89440	18g87680	18g87730
PPDK	Pyruvate, phosphate dikinase	Plastid	…	…	16g82470	16g82280	16g82490
PEPCK	Phosphoenolpyruvate carboxykinase	Cytosol	03p22140	03g22140	…	…	…
HCO3	Bicarbonate transporter	Plastid	01p09530	01g09400	01g07380	01g07410	01g07450
HCO3	Bicarbonate transporter	Cell membrane	10p60400	10g60610	10g61530	11g64980	10g61790
αCA	Alpha carbonic anhydrase	Extracellular	12p65340	12g65560	12g69320	05g38040	12g69590
γCA	Gamma carbonic anhydrase	Mitochondrion	14p71660	14g71860	14g77690	14g77780	14g77850
OAA	2-oxoglutarate/malate translocator	Plastid	03p23830	03g23820	01g04760	01g04770	01g04800
OAA	2-oxoglutarate/malate translocator	Plastid	08p50500	08g50750	01g08930	01g08940	01g09010
MDH	NADP-dependent malate dehydrogenase	Plastid	04p28680	04g28660	08g54050	09g57500	08g54280
ME	NADP-dependent malic enzyme	Plastid	06p45830	06g46070	08g51440	09g54990	08g51880
PEPT	Phosphoenolpyruvate/phosphate translocator	Plastid	10p60390	10g60600	02g18270	02g19760	02g19970
PEPT	Phosphoenolpyruvate/phosphate translocator	Plastid	07p50200	07g50450	10g60650	11g64130	10g60950
Central carbon metabolism (CB cycle)							
FBPase (class I)	Fructose-1,6-bisphosphatase, redox-sensitive	Plastid	02p16320	02g16270	02g13280	02g14760	02g14980
FBPase (class I)	Fructose-1,6-bisphosphatase, redox-insensitive	Plastid	…	…	19g88610	18g86850	18g86890
FBPase (class II)	Fructose-1,6-bisphosphatase, with GlpX domain	Plastid	06p44780	06g45010	01g06550	01g06560	01g06590
Central carbon metabolism (OPPP)							
G6PDH	Glucose-6-phosphate 1-dehydrogenase	Plastid	13p70070	13g70280	01g02140	01g02180	01g02160
G6PDH	Glucose-6-phosphate 1-dehydrogenase	Cytosol	…	…	18g87060	19g88730	19g88710
6PGL	6-phosphogluconolactonase	Plastid	13p67850	13g68030	01g00080	01g00080	01g00080
6PGD	6-phosphogluconate dehydrogenase	Plastid	07p47470	07g47690	10g63480	11g66850	10g63680
6PGD	6-phosphogluconate dehydrogenase	Cytosol	…	…	18g87680	19g89380	19g89360

Two *C*. *roscoffensis* genes encode distinct PEPCase proteins, i.e. a plant-like protein (EC 4.1.1.31) and a bacterial-type protein (EC 4.1.1.31), which like their plant orthologs presumably function as multisubunit complexes: a homotetramer (class-1 PEPCase) formed by the plant-type PEPCase and a heterooctamer (class-2 PEPCase) composed of the plant-type and bacterial-type PEPCase proteins (O'Leary et al. 2009; Park et al. 2012). Given that the class-2 PEPCase has been shown to associate with the surface of mitochondria in plants (Park et al. 2012), it has been proposed that this complex facilitates the capture of CO_2_ arising from the TCA cycle and photorespiration. A mitochondrial-targeted ɣ-type carbonic anhydrase (ɣCA) is also thought to participate in this function as part of a mitochondrial HCO_3_^−^ export system (Zabaleta et al. 2012; Soto et al. 2015). Proteins belonging to this CA subfamily, which are components of complex I (NADH:ubiquinone oxidoreductase) of the mitochondrial electron transport chain, have been identified in both *Chloropicon* species as well as in a variety of eukaryotes, excluding animals and fungi (Gawryluk and Gray 2010) (Fig. S4). Each of the analyzed *Chloropicon* genomes contains a single gene encoding ɣCA and the predicted protein features all critical residues that are required for catalysis, including the three Zn^2+^-binding histidine residues (Gawryluk and Gray 2010).

*Chloropicon primus* is lacking two of the aforementioned proteins: PPDK and the bacterial-type PEPCase (Table 2). Instead, it uniquely possesses an alternative C_4_ decarboxylating enzyme (PEPCK, PEP carboxykinase, EC 4.1.1.49) that directly transforms OAA into PEP concomitant with the release of a CO_2_ molecule; however, no plastid target peptide was detected in the predicted sequence of this protein, suggesting that it occurs in the cytosol. The *C. primus* PEPCK was probably acquired by horizontal gene transfer, as its closest homologs in BLASTP searches against the NCBI non-redundant protein database originate from bacteria (91% sequence similarity with a PEPCK from a *Longimicrobiales* bacterium).

#### Central Carbon Metabolism

Relative to *C. primus*, *C*. *roscoffensis* features additional versions of proteins involved in the CB cycle and the oxidative phase of the pentose phosphate pathway (OPPP) (Table 2). During photosynthesis, fructose-1,6-bisphosphatase (FBPase, EC 3.1.3.11) participates in the CB cycle by catalyzing the irreversible breakdown of fructose-1,6-bisphophate into fructose-6-phosphate and P_i_, a reaction that takes place in the chloroplast. In addition to this key enzyme of the CB cycle, which is light-activated via a ferredoxin-linked thioredoxin system (the enzyme is activated when a regulatory disulfide bridge in the active site is reduced by thioredoxin *f)* (Balsera et al. 2014), *C*. *roscoffensis* contains two other chloroplast-targeted FBPases (Table 2). One of these isoforms, usually found in non-green algae and streptophytes, also belongs to class I although it acts independently of redox activation (lacking the domain associated with redox regulation) (Li et al. 2020b). The second isoform features a GlpX domain (class-II member) and its homologs occur mostly in bacteria and in some core chlorophytes. *Chloropicon primus*, however, contains only two chloroplast-targeted FBPases, missing the redox-insensitive class-I enzyme found in *C*. *roscoffensis*. None of the examined *Chloropicon* taxa was found to possess a cytosolic FBPase, an isoform commonly found in land plants.

The OPPP generates ribulose-5-phosphate from glucose-6-phosphate with the concomitant reduction of NADP^+^ to NADPH; the resulting ribulose-5-phosphate is used for nucleic acid synthesis, among other things, while the NADPH is needed for several anabolic reactions and for ROS scavenging systems (Sharkey 2021). As observed for all other photosynthetic eukaryotes, the examined *Chloropicon* isolates harbor in their chloroplast the three enzymes involved in this pathway (Table 2). In addition, cytosolic isoforms of two of these enzymes, i.e. glucose-6-phosphate 1-dehydrogenase (G6PDH, EC 1.1.1.49) and 6-phosphogluconate dehydrogenase (6PGD, EC 1.1.1.44), are present in *C*. *roscoffensis*, but not in *C*. *primus.*

### *Chloropicon* Genes Involved in Queuosine Metabolism

Among the proteins shared by *C*. *primus* and *C*. *roscoffensis* but missing in most or all other green algae, we identified four that are involved in queuosine (Q) metabolism. Widespread in eukaryotes and bacteria, Q is a modified nucleoside found at the wobble position of tRNAs with GUN anticodons (tyrosyl-, aspartyl-, asparaginyl-, and histidyl-tRNAs). This complex tRNA modification plays an important role in the efficiency and accuracy of protein synthesis. Most bacteria synthesize Q de novo, using a multistep pathway that starts with the synthesis of the 7-aminomethyl-7-deazaguanine base intermediate (preQ_1_) from GTP, followed by the exchange of preQ1 with guanine at position 34 of the target tRNA by a bacterial-type tRNA guanosine transglycosylase (bTGT, EC 2.4.2.29), which exists as a homodimer, and finally by two enzymatic steps catalyzed by QueA (tRNA preQ_1_ (34) S-adenosylmethionine ribosyltransferase-isomerase, EC 2.4.99.17) and QueG (tRNA epoxyqueuosine (34) reductase, EC 1.17.99.6) (Hutinet et al. 2017). Numerous bacteria have lost the genes necessary for preQ_0_ and preQ_1_ synthesis; they likely produce Q-modified tRNAs by obtaining precursors via a salvage pathway (Iwata-Reuyl and de Crécy-Lagard 2009). Conversely, all the eukaryotes investigated so far appear to strictly depend on salvage of queuine (q), the base of Q, directly from the environment (microbiota and/or ingested food) or alternatively on its recycling from precursors generated by degradation of Q-modified tRNAs (Zallot et al. 2014). Both q and Q are incorporated into the eukaryotic cell via unknown transporters, but only q can be inserted directly into target tRNAs using the eukaryotic type tRNA-guanine transglycosylase (eTGT, EC 2.4.2.29), which is a heterodimer composed of a catalytic subunit (QTRT1) and a regulatory subunit (QTRT2, previously called QTRTD1). Q must therefore be converted to q prior to incorporation of the latter base into tRNA; the Q-nucleotide N-glycosylase/hydrolase (QNG1 for the mammalian enzyme and Qng1 for the bacterial ortholog), formerly known as DUF2419 (Zallot et al. 2014), catalyzes this reaction using Q-5′-monophosphate as primary substrate (Hung et al. 2023).

**Fig. 5.**
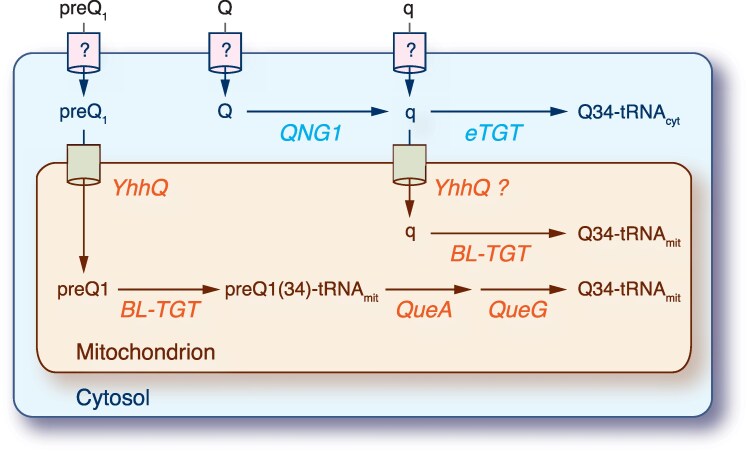
Proposed model for Q salvage and tRNA queuosine modification of *Chloropicon* cytosolic and mitochondrial tRNAs with GUN anticodons. BL-TGT, bacterial-like queuine tRNA-ribosyltransferase; eTGT, eukaryotic queuine tRNA-ribosyltransferase; QNG1, queuosine-nucleotide N-glycosylase/hydrolase; QueA, tRNA preQ_1_(34) S-adenosylmethionine ribosyltransferase-isomerase; QueG, tRNA epoxyqueuosine(34) reductase; YhhQ, queuosine preQ_0_/preQ_1_ precursor transporter.

In all isolates of *C. primus* and *C. roscoffensis*, we identified QNG1 and both subunits of the eTGT complex as well as three putative bacterial-type proteins involved in Q synthesis, hereafter designated as BL-TGT (Bacterial-Like TGT, EC 2.4.2.29), QueA, and QueG (Table 3; Fig. 5). In addition, the bacterial-type YhhQ transporter (COG1738) for preQ_0_/preQ_1_ was detected in both *C. primus* isolates and in *C. roscoffensis* RCC2335. Interestingly, the genes coding for QueA and YhhQ are physically linked on chromosome 17 of *C*. *primus*, whereas they reside on distinct chromosomes (9 and 17, respectively) in *C*. *roscoffensis* RCC2335. Analysis of protein subcellular localization revealed that YhhQ, BL-TGT, QueA, and QueG are targeted to mitochondria, whereas the three other proteins are cytosolic (Table 3; Fig. 5).

**Table 3:** *Chloropicon* proteins involved in Q metabolism

Protein	Function	Localization	*Chloropicon primus*		*Chloropicon roscoffensis*		
			CCMP1205	RCC138	RCC1871	RCC2335	CCMP1998
QTRT1	Catalytic subunit of eukaryotic queuine tRNA-ribosyltransferase	Cytosol	05p40030	05g40100	05g39250	04g32220	05g40010
QTRT2	Accessory subunit of eukaryotic queuine tRNA-ribosyltransferase	Cytosol	06p42550	06g42800	03g20890	03g22430	04g30180
QNG1	Queuosine-nucleotide N-glycosylase/hydrolase	Cytosol	13p69450	13g69650	01g01530	01g01530	01g01540
BL-TGT	Bacterial-like queuine tRNA-ribosyltransferase	Mitochondrion	08p52200	08g52500	04g32770	08g51330	03g26280
YhhQ	Queuosine preQ_0_/preQ_1_ precursor transporter	Mitochondrion	17p79880	17g80100	…	09g55700	…
QueA	tRNA preQ_1_ (34) S-adenosylmethionine ribosyltransferase-isomerase	Mitochondrion	17p79870	17g80090	17g85160	17g84980	17g85130
QueG	tRNA epoxyqueuosine (34) reductase	Mitochondrion	17p79600	17g79820	17g85480	17g85290	17g85440

We performed BlastP searches against the NCBI non-redundant protein database using as queries the seven aforementioned proteins of *C*. *primus* or *C. roscoffensis* in order to identify their homologs in other organisms and compare their phylogenetic distributions. As expected, the ubiquitous cytosolic proteins QTRT1, QTRT2, QNG1 clustered with green algal and land plant orthologs in phylogenetic trees (Fig. S5a–c). With regards to the analysis of the YhhQ transporter, our BlastP searches returned exclusively bacterial proteins, whereas both bacterial and eukaryotic proteins were identified as homologs of *Chloropicon* BL-TGT, QueA, and QueG, with the closest relatives of the latter proteins coming from red algae (Fig. S5d–g). As indicated by the phylogenetic distribution of their homologs, the *Chloropicon* BL-TGT proteins belong to a subgroup of TGT enzymes that have been identified in diverse algae and protozoa (Manna and Harman 2016). Localized mostly in the mitochondrion, these eukaryotic proteins were originally acquired from bacteria of the *Chlamydia* lineage (Manna and Harman 2016).

The above results indicate that like other eukaryotes, all *C. primus* and *C. roscoffensis* isolates can import Q or q from their extracellular environment or alternatively can salvage Q derived from the degradation of intracellular Q-modified tRNAs to generate newly synthesized Q-modified tRNAs in the cytosol via the eTGT complex (Fig. 5). In addition, our analyses predict that both *C. primus* isolates and *C. roscoffensis* RCC2335 can import preQ_1_ from their environment via an unknown transporter and subsequently transfer it to the mitochondria via the YhhQ transporter where this precursor is transformed into Q-modified tRNA using BL-TGT, QueA and QueG. For the *C. roscoffensis* isolates lacking YhhQ, we suggest that like their Chlamydial homologs (Yuan et al. 2019), the BL-TGT enzymes of these strains are able to use q as substrate.

## Discussion

For picoplanktonic marine green algae, little is known about their life cycle and the genomic changes that occurred during speciation or adaptation to new oceanic environments. The genomes of the five *Chloropicon* strains analyzed in this study, two *C*. *primus* and three *C. roscoffensis* isolates, provide insights into the evolutionary changes that occurred during divergence of the closely related *C*. *primus* and *C*. *roscoffensis* from their common ancestor as well as following the birth of the *C*. *roscoffensis* species. While the observed genomic changes did not correspond to substantial alterations in overall gene content, we identified distinct gene differences associated with well-characterized cellular functions. Specifically, *C. roscoffensis* was found to possess genes implicated in the CCM, the CB cycle, and the OPPP. Furthermore, our analysis uncovered a previously unreported bacterial-type queuosine (Q) salvage and tRNA modification pathway localized within the mitochondria, suggesting a novel aspect of mitochondrial RNA processing in this species.

### Intra- and Interspecific Genomic Variations Observed for *C*. *primus* and *C*. *roscoffensis*

Our results revealed that the *C*. *primus* genomes differ from their *C*. *roscoffensis* counterparts at the levels of chromosome number (20 vs. 19), ploidy state (predominantly diploid vs. haploid/diploid), gene content, and overall gene arrangement on nearly half of the chromosomes (Table 1; Figs. 1–3). As expected, intraspecific differences proved to be much less conspicuous. The genomes of the *C*. *primus* isolates, which were isolated from different oceans (Table 1), are both predominantly diploid, with one or two chromosomes present in three copies (chr 17 in CCMP1205 and RCC138 and chr 20 in RCC138), and despite the presence of a moderate level of heterozygous sites, perfect collinearity was observed for all 20 chromosomes. Considering that aneuploidy has been shown to arise frequently in cultures of Mamiellophyceae (Krasovec et al. 2023) and that the *C*. *primus* strains analyzed in the present study were maintained in cultures for many years after their isolation (Table 1), it is possible that *C*. *primus* is completely diploid in natural environments. Whereas the genomes of the examined *C*. *roscoffensis* strains displayed no aneuploid chromosomes, they varied in ploidy state, with the Atlantic isolate from the Sargasso Sea and the Pacific isolate from the Sagami Bay being both haploid and the Atlantic isolate from the English Channel being diploid. This ploidy change is consistent with the approximately two-fold variation in genome size previously estimated for the RCC2335 and RCC1871 strains (about 20 and 40 Mb, respectively) using flow cytometry (Lopes dos Santos et al. 2017b). The level of heterozygosity of the diploid RCC1871 strain was much higher than those observed for the *C*. *primus* strains (Table 1) and additionally, contrary to the CCMP1205 and RCC138 strains, large-scale rearrangements of a few chromosomes were identified between the *C*. *roscoffensis* strains (Fig. 2). Notably, the two isolates from the Atlantic Ocean displayed the least number of rearrangements, suggesting a possible link between genome divergence and geographical location.

To our knowledge, this comparative analysis of *C. roscoffensis* genomes provides the first documented case of a picoplanktonic marine green alga exhibiting distinct haploid and diploid vegetative cell forms. The presence of multiple ploidy states in this species may confer several adaptive advantages, as suggested by analogous findings in other organisms, particularly yeasts. Studies in yeast have proposed several benefits of haploidy, including faster adaptation to novel environments due to the immediate expression and selection of beneficial mutations. Haploids may also exhibit enhanced tolerance to environmental stress and improved growth under nutrient-limited conditions, potentially due to a higher surface area-to-volume ratio—a trait observed in the RCC2335 strain of *C. roscoffensis* compared to the RCC1871 strain (Lopes dos Santos et al. 2017b). On the other hand, diploidy may offer greater genetic flexibility and increased resistance to large-scale genomic rearrangements (Gerstein et al. 2011; Zorgo et al. 2013; Sharp et al. 2018). In other marine algae, such as the coccolithophore *Gephyrocapsa huxleyi* (formerly *Emiliania huxleyi*), a haplodiplontic life cycle with distinct morphologies has been hypothesized to facilitate adaptation to diverse ecological niches (de Vries et al. 2021). In the dinoflagellate *Durusdinium trenchii*, a facultative coral symbiont, it has been shown that whole-genome duplication (polyploidization) is associated with enhanced stress tolerance—particularly thermal stress— and may facilitate its transition to a symbiotic lifestyle (Dougan et al. 2024). By analogy, the ability of *C. roscoffensis* to alternate between haploid and diploid states may enhance its ecological plasticity, enabling it to thrive in a broader range of oceanic environments. Supporting this hypothesis, a biogeographical study of the Chloropicophyceae revealed that the *C. roscoffensis* lineage is dominant in oceanic waters and has been isolated across wide latitudinal and oceanic ranges (Lopes dos Santos et al. 2017a).

Because sexual reproduction has not been observed for the Chloropicophyceae, it remains unknown whether *C*. *roscoffensis* is capable of switching between ploidy states through gametic fusion of two haploid cells and/or through meiosis of a diploid cell. The capacity to switch between ploidy states is a likely possibility considering that large sets of genes involved in meiosis and flagellar formation have been identified in the *C*. *primus* genome (Lemieux et al. 2019). An alternative way to switch from haploidy to diploidy would be through genome duplication without mitotic cell division, a phenomenon called endoreplication. Considering the high level of heterozygosity of the *C*. *roscoffensis* RCC1871 strain, however, it would seem more likely that the diploid state of this alga was the result of gametic fusion between distinct haploid cells rather than genome duplication.

Unlike the Chloropicophyceae, all strains of the Mamiellophyceae whose genomes have been reported proved to be haploid, and there is good evidence supporting this notion. In this context, an interesting question is how variable is the ploidy level among chlorophytes and whether a specific ploidy state is prevalent. Unfortunately, at this time, it is difficult to provide a firm answer, as numerous green algal genomes described in the literature were not sequenced using techniques and bioinformatic methodologies that enabled full genome assembly and the determination of ploidy level. But among the recently published chlorophyte genomes that were characterized with improved methodologies, it is noticeable that several are diploid, including the genomes of *Tetraselmis helgolandica* (Chlorodendrophyceae) (Zhou et al. 2025), *Picochlorum* species (Trebouxiophyceae) (Foflonker et al. 2018; Becker et al. 2020; da Roza et al. 2024), and representatives of the Chlorophyceae (Bogen et al. 2013; Biondi et al. 2024; Marcolungo et al. 2024). Notably, as observed in our study, marked differences were observed at the heterozygosity level for *Picochlorum* species (Foflonker et al. 2018).

### *Chloropicon roscoffensis*-Specific Proteins Associated With the CCM, CB Cycle, and OPPP

#### CCMs

As the demand for CO_2_ in photosynthesis exceeds the concentration of dissolved CO_2_ in seawater, CO_2_ can be an important factor limiting the proliferation of marine algae. In response to the restricted availability of dissolved CO_2_, aquatic photosynthetic eukaryotes evolved CCMs to maximize CO_2_ accumulation around Rubisco and enhance growth rates (He et al. 2023). CCMs can be divided into two broad classes: biophysical CCMs that use only HCO_3_^−^ as the intermediate molecule to concentrate CO_2_ and biochemical CCMs that transiently fix CO_2_ into intermediate organic molecules such as OAA and malate. Biophysical CCMs appear to be the dominant pathway for carbon fixation in chlorophytes and most of them occur in the pyrenoid, a structure that is lacking in *Chloropicon* species (Lopes dos Santos et al. 2017b). Here, we present evidence, based on gene annotation and predicted protein subcellular localization, that *C. roscoffensis* possesses a biochemical C_4_-like CCM (Fig. 4), closely resembling the theoretical model previously proposed for *Micromonas commoda, O. tauri,* and *Prasinoderma coloniale* (Worden et al. 2009; Li et al. 2020a). Although traditionally associated with land plants, biochemical C_4_ or C_4_-like CCMs have been documented for a few other green algae such as the marine macroalgae *Udotea flabellum* and *Ulva prolifera* (Ulvophyceae), as well as for the marine diatom *Thalassiosira weissflogii* (Liu et al. 2020). Interestingly, the C_4_-like CCM of *Ulva prolifera*, which involves PEPCK as the decarboxylating enzyme (instead of ME as found in *C*. *roscoffensis*), operates in conjunction with a biophysical CCM and these two CCMs are modulated differently depending on environmental conditions (Liu et al. 2020).

Our data predict that the biochemical CCM of *C*. *primus* differs from that of *C*. *roscoffensis* with respect to the C_4_ decarboxylating enzyme, but its complete pathway could not be reconstructed without any ambiguity. In *C. primus*, the unique presence of PEPCK, a protein likely gained from bacteria through horizontal transfer, was found to be correlated with the loss of PPDK (the enzyme converting pyruvate into PEP), raising the possibility that this alga might use a PEPCK of bacterial origin to directly decarboxylate OAA into PEP. This hypothesis would resolve the problem posed with the missing PEP-regenerating PPDK; however, bioinformatics analysis using DeepLoc 2.1 and PredAlgo failed to detect the presence of a plastid target signal in the *C*. *primus* PEPCK. Note here that a biophysical CCM is unlikely to participate in CO_2_ assimilation in *Chloropicon* species, given that no plastid-targeted CA was detected.

An interesting aspect of the *C*. *roscoffensis* biochemical CCM model is its association with a pathway that may enable the recycling of mitochondrial CO_2_ for carbon fixation in the chloroplast (Fig. 4). This pathway, which prominently involves mitochondrial-targeted ɣ-type CAs integrated into complex I of the mitochondrial electron transport chain, has been postulated to be part of the basal biochemical CCM in land plants (Zabaleta et al. 2012). Mitochondrial CO_2_ recycling for carbon fixation has not been previously documented for green algae but appears to be widespread among chlorophytes, as indicated by our BlastP searches using the participating proteins of *Chloropicon* (ɣCA and PEPCases). The ɣCA domain of complex I is attached to the membrane arm on the matrix side of the inner mitochondrial membrane and near this domain there is a bridge connecting the membrane arm to the Q domain of the peripheral arm (Klusch et al. 2021). Given the presence of a unique ɣCA-encoding gene in *Chloropicon* species, the ɣCA domain of these picoalgae is predicted to be a homotrimer. The ɣCA domain is essential for assembly of the protein complex, and in photosynthetic eukaryotes, it is thought to be also required for transfer of mitochondrial CO_2_ to the chloroplast for carbon fixation by the CB cycle (Zabaleta et al. 2012; Klusch et al. 2021). Considering that the high-resolution cryoEM structures determined for complex I from *Arabidopsis thaliana* do not support the notion that this plant complex is directly involved in CO_2_ or HCO_3_^−^ transport across the inner mitochondrial membrane (Klusch et al. 2021); HCO_3_^−^ formed at the heterotrimeric ɣCA domain may be exported from the mitochondrial matrix by transporters unrelated to complex I. Furthermore, it is tempting to speculate that interactions between structural domains in complex I and disordered regions of bacterial-type PEPCase provide an interface between mitochondrial respiration and CO_2_ recycling (Park et al. 2012).

#### Central Carbon Metabolism

The *C*. *roscoffensis*-specific proteins participating in the CB cycle and OPPP suggest that this species is better able to cope with abiotic stresses than *C*. *primus*. The *C*. *roscoffensis* chloroplast features three isoforms of FBPase, a key regulatory enzyme of the CB pathway. Two isoforms are shared with *C. primus*, but a third one is found only in *C. roscoffensis*. The latter isoform has no regulatory redox domain and is more tolerant to oxidative stress (Serrato et al. 2009; Ogawa et al. 2015). It has been suggested that the coexistence of this redox-insensitive isoform along with the redox-sensitive isoform of the same enzymes in the chloroplasts of diverse algae and land plants is the consequence of adaptive evolution to cope with fluctuating redox conditions (Ogawa et al. 2015; Li et al. 2020b). For instance, the redox-insensitive isoform is thought to participate in the CB cycle under abnormal oxidative conditions caused by low light and low temperature (Li et al. 2020b).

Among the enzymes known to participate in the OPPP, the cytosolic isoforms of G6PDH and 6PGD were identified in *C*. *roscoffensis* but not in *C*. *primus* (Table 2). Cytosolic isoenzymes catalyzing part or all three OPPP reactions have also been detected in plants (Sharkey 2021) and other green algae, including *Micromonas*, *Bathycoccus,* and trebouxiophytes. Although few studies have been published about G6PDH in algae, it is clear that, as in land plants, this key enzyme of the OPPP is involved in the supply of reducing power (NADPH) and precursors for reductive biosynthetic reactions (e.g. assimilation of inorganic nitrogen, fatty acid synthesis) (Esposito 2016; Jiang et al. 2022). In addition, it plays a central role in counteracting the effects of stress by providing reductants for ROS scavenging and maintenance of redox balance. For land plants, it has been shown that cytosolic and plastid G6PDHs are part of a broader response to various types of stresses, including low temperature, drought, freezing, oxidative stress, and potentially other environmental challenges (high salt, heavy metal) (Jiang et al. 2022). The studies that examined both G6PDH and 6PGD generally observed parallel responses, suggesting a synergistic role in plant response to various stresses (Tian et al. 2021).

### Discovery of a Bacterial-type Q Salvage and tRNA Queuosine Modification in *Chloropicon* Mitochondria

The physiological roles of tRNA queuosine modification have been recently elucidated in both bacteria and eukaryotes, thus bolstering the importance of this process, which is critical for the efficiency and fidelity of mRNA translation (Diaz-Rullo and Gonzalez-Pastor 2023; Diaz-Rullo et al. 2025). However, little is known about q modification of tRNAs in organelles. In most green algae, all four tRNAs with GUN anticodons (tyrosyl-, aspartyl-, asparaginyl- and histidyl-tRNAs) are encoded on both the chloroplast and mitochondrial genomes (Turmel et al. 2013; Turmel and Lemieux 2018), but to our knowledge, these organelle tRNAs have not been experimentally shown to be modified with q in any algae. Even in mammalians where q-modified tRNAs have been documented, the mechanism responsible for their modification is still unclear (Boland et al. 2009). Our study is the first to report evidence for the existence of a bacterial-type Q salvage and tRNA modification pathway in mitochondria. Our data suggest that both *C. primus* isolates and *C. roscoffensis* RCC2335 are able to transport preQ_1_ to the mitochondria via YhhQ and transform tRNA with GUN anticodons into Q-modified tRNAs using enzymes known to participate in q de novo synthesis in bacteria (BL-TGT, QueA, and QueG) (Fig. 5). This mitochondrial salvage pathway likely has an ancient origin, potentially dating back to the origin of the Archaeplastida or even to early eukaryotes. Supporting this, some red algae—most notably *Galdieria sulphuraria*—possess mitochondrial-targeted BL-TGT, QueA and QueG, which cluster with *Chloropicon* orthologs in phylogenetic trees. This ancestral mechanism, however, has been simplified early during green plant evolution, as YhhQ, QueA, and QueG are absent in almost all extant green plants. By allowing the use of preQ_1_ in addition to Q and q precursors, the retention of this ancestral pathway likely enhances the potential of *Chloropicon* species to survive in nutrient-poor environments.

Notably, *C*. *roscoffensis* CCMP1998 and RCC1871 are missing the YhhQ-encoding gene (Table 3). The impact of this gene loss remains unclear. If preQ_1_ uptake depends on the YhhQ transporter, the presence of mitochondrial QueA and QueG may be dispensable, suggesting their nuclear-encoded genes could be lost over time. Knowledge about the substrate selectivity of green algal BL-TGT proteins is also critical for evaluating the consequence of the absence of YhhQ in *C*. *roscoffensis* CCMP1998 and RCC1871. The presence of mitochondrial-targeted BL-TGT in some green algae lacking QueA and QueG (Manna and Harman 2016) suggests that BL-TGT can catalyze the exchange of guanine for q on newly synthesized mitochondrial tyrosyl-, aspartyl-, asparaginyl-, and histidyl-tRNAs with GUN anticodons. Importantly, it has been experimentally demonstrated that the intracellular pathogen *Chlamydia trachomatis*, which is lacking QueA or QueG/H, is able to import q using its YhhQ transporter and then insert it into tRNA using its BL-TGT enzyme (Yuan et al. 2019). The determinants of substrate specificity for the bTGT, eTGT, and BL-TGT enzymes reside in the substrate-binding pocket (Stengl et al. 2005; Chen et al. 2011; Biela et al. 2013), and given that the amino acid differences observed for the *C*. *trachomatis* BL-TGT binding pocket are also shared by all other members of the BL-TGT group, including the *Chloropicon* proteins, it is plausible that all BL-TGTs are able to catalyze the base exchange between q and the target guanine in tRNA. Independent support for this hypothesis comes from functional studies of mutated bTGT and eTGT enzymes and the recent finding that the human eTGT displays promiscuous nucleobase preference (Fergus et al. 2021). In the bTGT of *Zymomonas mobilis*, a Val233Gly change has been shown to enlarge the substrate binding pocket, allowing it to accommodate q without using it as a substrate (Biela et al. 2013). A Gly residue is found at the corresponding positions of the human eTGT (position 232) and all BL-TGTs (position 307 in *Chloropicon*). Moreover, a Cys158Val change of the *Z*. *mobilis* enzyme is known to reduce its affinity to preQ_1_, while leaving the affinity to guanine unaffected (Biela et al. 2013), and a Leu residue is present at the corresponding positions of BL-TGTs (position 235 in *Chloropicon*). Provided that the retention of YhhQ enables *C*. *primus* and *C*. *roscoffensis* RCC2335 to use preQ_1_ in addition to Q and q, perhaps this capability may enhance the accuracy of mitochondrial mRNA translation in these strains compared to *Chloropicon* strains lacking the transporter.

## Conclusion

The genomic data and analyses reported in this study contribute to our understanding of the evolutionary dynamics and ecological success of *Chloropicon* species in marine environments. More specifically, they shed light on the genomic variations displayed by the closely related *C*. *primus* and *C*. *roscoffensis* species and report the existence of a bacterial-type Q salvage and tRNA modification pathway in *Chloropicon* mitochondria. Despite their many shared similarities, the *C*. *primus* and *C*. *roscoffensis* genomes differ mostly in the organization of a number of their chromosomes and in their ploidy states. While the two examined *C*. *primus* species are predominantly diploid, two of the three *C*. *roscoffensis* species are haploid and the third is diploid. The maintenance of both haploid and diploid populations may confer advantages to *C*. *roscoffensis*, particularly by enhancing its ability to adapt to diverse oceanic environments. This could help explain why the *C*. *roscoffensis* lineage is among the most dominant *Chloropicon* lineages in oceanic waters. In addition, we suggest that the functions of the CCM, FBPase, and OPPP genes that have not been retained in *C*. *primus* may contribute to increased metabolic flexibility and better survival of *C*. *roscoffensis* in different oceanic regions by providing a more efficient biochemical CCM and promoting more tolerance to environmental stresses. To evaluate these hypotheses, it will be interesting to look at the genomes of other *Chloropicon* lineages in future studies.

## Materials and Methods

### Cell Culture and DNA/RNA Extraction

*Chloropicon roscoffensis* CCMP1998 (clade VII A4; isolated from the Sargasso Sea in 1998) was obtained from the Provasoli-Guillard National Center for Marine Algae and Microbiota (East Bootbay, ME, USA). *Chloropicon primus* RCC138 (clade VII A2; isolated from the North Pacific Ocean in 1992), *C. roscoffensis* RCC1871 (clade VII A4; isolated from the English Channel in 2009), and *C. roscoffensis* RCC2335 (clade VII A4; isolated from Sagami Bay, Japan in 2009) were obtained from the Roscoff Culture Collection (RCC) of the Station Biologique de Roscoff (Roscoff, France).

*Chloropicon* cells were grown in L1 medium at 18 °C under alternating 12 h light/dark periods of Cool White fluorescent illumination. Cells were collected by centrifugation at the onset of the light period and ground in liquid nitrogen using a mortar and pestle. Ground cells were resuspended in 50 mM Tris-HCl pH 8.0, 20 mM EDTA, 0.8 M NaCl, 1% SDS, 1% CTAB, 0.5% PVP 40,000, 10 mg/mL proteinase K and incubated for 30 min at 65 °C. DNA was extracted from each lysate once with an equal volume of phenol:chloroform:isoamyl alcohol 25:24:1 and twice with an equal volume of chloroform:isoamyl alcohol 24:1, then precipitated with two volumes of cold 99% ethanol at −20 °C for 30 min. RNA samples were extracted using the E.Z.N.A. Total RNA kit II from Omega Bio-Tek (Norcross, GA, USA) and their integrity was monitored using a Bioanalyzer instrument from Agilent Technologies (Santa Clara, CA, USA).

### DNA and RNA Sequencing

DNA preparations were sequenced using a combination of short and long reads generated on Illumina (San Diego, CA, USA), PacBio (Menlo Park, CA, USA), and Oxford Nanopore (Oxford, United Kingdom) platforms (Tables S2 and S3). Paired-end Illumina libraries were prepared using the Illumina TruSeq DNA kit and sequenced on Illumina MiSeq or HiSeq 4000 instruments at the Plateforme d’Analyses Génomiques of Université Laval (Québec, QC, Canada) or the McGill University and Génome Québec Innovation Centre (Montréal, Canada), respectively. Nanopore libraries were prepared using the Ligation Sequencing kit V9 from Oxford Nanopore, sequenced in-house in the Turmel/Lemieux laboratory on a MinION MN25315 instrument, and basecalled with GUPPY 3.1.5 on NVIDIA 16G Tesla-P100 GPUs. PacBio sheared genomic DNA (gDNA) large insert libraries were prepared and sequenced on a Sequel instrument at Génome Québec. RNAseq libraries were constructed using the NEBNext ULTRA II Directional RNA kit from New England Biolabs (Ipswich, MA, USA) and sequenced on an Illumina HiSeq 4000 instrument at Génome Québec.

### Genome Assemblies

Illumina sequencing datasets were quality-checked with FastQC 0.12.1 (https://www.bioinformatics.babraham.ac.uk/projects/fastqc/). Low-quality bases and adapter sequences in the Illumina reads were removed using AfterQC 0.9.7 (Chen et al. 2017). *Chloropicon roscoffensis CCMP1998:* PacBio Sequel and Illumina HiSeq 4000 reads were independently assembled using Canu 1.8 (Koren et al. 2017) and SPAdes 3.13.0 (Prjibelski et al. 2020), respectively. These assemblies were merged using the alignment tool for large DNA fragments implemented in Sequencher 5.4.1 (Gene Codes Corporation, Ann Arbor, MI, USA). Discrepancies in the consensus sequence were resolved manually using the Sequencher editing tool and the basecalling of the Illumina contigs. *Chloropicon roscoffensis RCC2335:* PacBio Sequel and Oxford Nanopore MinION reads were assembled using Canu 1.8, and the consensus sequences of the resulting contigs were polished with Illumina MiSeq data iteratively for ten cycles with Pilon 1.23 (Walker et al. 2014). *Chloropicon roscoffensis RCC1871:* PacBio Sequel reads were assembled using the Falcon-unzip module from the PacBio Assembly Tool Suite (May 2020 version). Assembled contigs were first polished with PacBio data using the Arrow module from the PacBio Assembly Tool Suite, then with Illumina MiSeq reads iteratively for four cycles with Pilon 1.23. Because of the diploid and highly heterozygous state of the *C. roscoffensis* RCC1871 genome, the Falcon-unzip assembly yielded 101 haplotigs in addition to the primary Telomere-to-Telomere (T2T) chromosomes. *Chloropicon primus RCC138:* Illumina MiSeq reads were assembled using SPAdes 3.13.0. Oxford Nanopore reads were assembled with Canu 1.8, and the resulting contigs were polished first with Oxford Nanopore reads using Nanopolish 0.11.0 (https://github.com/jts/nanopolish), then with the Illumina MiSeq reads using four iterative rounds with Pilon 1.23. The Nanopolish/Pilon polished chromosome sequences were compared visually against the SPAdes contigs using Sequencher 5.4.1 and remaining discrepancies in the consensus sequences were curated manually. Chromosome completeness and assembly metrics of the *Chloropicon* genomes were assessed/calculated with check_for_telomeres.pl 0.4b (this study) using the hexamer TTTAGG as telomeric pattern query.

### Genome Annotations

Transfer and ribosomal RNA genes in the *Chloropicon* genomes were identified using tRNAscan-SE 2.0.4 (Chan et al. 2021) and RNAmmer 1.2 (Lagesen et al. 2007), respectively. Protein-coding genes were predicted independently using the MAKER 3.01.03 (Campbell et al. 2014 ) and BRAKER 2.1.5 (Bruna et al. 2021) pipelines. The *C. primus* RCC138 genome was annotated as follows. MAKER predictions (leveraging Augustus 3.3.3 (Stanke et al. 2008)) were performed using the *C. primus* CCMP1205 gene model from Lemieux et al. (2019). BRAKER predictions were performed using the *C. primus* CCMP1205 RNAseq data (Lemieux et al. 2019) (Tables S2 and S3) filtered with fastp 0.20.0 (Chen et al. 2018) using a minimum length of 100 nt and a mean cut quality score of 30, then mapped onto the RCC138 genome with HISAT2 2.1.0 (Kim et al. 2019) using a maximum intron length of 5,000 nt. Gene predictions were annotated and curated with Apollo 2.5.0 (Dunn et al. 2019) and the A2GB pipeline (https://github.com/PombertLab/A2GB) as described in Mascarenhas dos Santos et al. (2023). Protein functions were inferred from InterProScan5 5 + searches (Jones et al. 2014) and from sequence homology searches with DIAMOND 2+ (Buchfink et al. 2021) against UniProt's SwissProt/TrEMBL databases (UniProt Consortium 2023) and against *C. primus* CCMP1205 annotations from Lemieux et al. (Lemieux et al. 2019). Protein functions inferred from these analyses were curated with curate_annotations.pl from A2GB. The *C. roscoffensis* RCC1871, RCC2335, and CCMP1998 genes were annotated as described above, except that BRAKER predictions were performed by leveraging distinct NCBI SRA RNAseq datasets (Tables S2 and S3).

Completeness of the *C. primus* and *C. roscoffensis* genome annotations was assessed with BUSCO 5.6.1 (Manni et al. 2021) using the chlorophyta_odb10 reference set (Fig. S6). Annotations metrics were calculated with gbff_parser.pl 0.1a (this study) from the corresponding GenBank (.gbff) files. Subcellular localization of proteins involved in selected metabolic pathways were predicted using DeepLoc 2.1 (Odum et al. 2024) and PredAlgo (Tardif et al. 2012).

### Repeats and Ploidy Analyses

Repeats in the *Chloropicon* genomes were searched for and masked with RepeatModeler 2.0.5 (Flynn et al. 2020) and RepeatMasker 4.1.5 (Smit et al. 2013-2015), respectively. Ploidies in the *Chloropicon* genomes were assessed by read mapping, variant calling, and plotting of the allelic frequencies of the inferred heterozygous loci, as follows. Low quality base calls in the Illumina paired end datasets were filtered out with fastp 0.23.4 (Chen et al. 2018) using a mean cut quality score of 30 and a minimum read length of 125 nt. FASTP-filtered Illumina reads were mapped in paired end mode against their respective masked genomes with minimap2 2.26 (Li 2021), SAM/BAM files were converted with samtools 1.18 (Danecek et al. 2021) and variants were called using VarScan2 2.4.6 (Koboldt et al. 2012), as implemented in get_SNPs.pl v2.0f (SSRG) from the SSRG pipeline (https://github.com/PombertLab/SSRG) using a minimum variant frequency of 0.1. Allelic frequencies were plotted from the resulting variant calling (.vcf) files using R 4.3.1 (R_Core_Team 2024): VCF files were split per chromosome with split_VCF.pl 0.2 (this study), SNPs were parsed with sort_SNPs.pl 0.2c (this study), and allelic frequencies were plotted with plot_r.pl 0.1 (this study). Aneuploid chromosomes detected from the R plots were further investigated by searching for deviations from average sequencing depths in the corresponding files generated by get_SNPs.pl 2.0f (SSRG) from the output of samtools depth -aa function.

### Comparative Genome Analyses

Orthologs shared between the *Chloropicon* genomes were searched for with OrthoFinder 2.5.5 (Emms and Kelly 2019), lists of orthogroups found in each species were generated with parse_OGs.pl 0.1 (this study), and the Venn diagram was generated using the online tool from the Bioinformatics & Evolutionary Genomics group of the University of Gent (https://bioinformatics.psb.ugent.be/webtools/Venn/). Orthogroups unique to each species were retrieved with get_species_set.pl 0.2 (this study). Orthogroup expansions/contractions were investigated with CAFE 5.1 (Mendes et al. 2020) using default parameters and as input, the OrthoFinder species tree (SpeciesTree_rooted.txt) made ultrametric with R 4.3.3 (R_Core_Team 2024) using the chronos function from the Analyses of Phylogenetics and Evolution (APE) library (Paradis et al. 2004) and the Orthogroups.GeneCount.tsv file reformatted for CAFE with og_to_cafe.pl 0.1 (this study). Significant changes labelled by asterisks in the CAFE asr.tre file were further investigated with cafe_sig_tree.pl 0.1a (this study).

Phylogenetic relationships among *Chloropicon* species were inferred using an amino acid dataset of 7,281 single-copy orthologs shared between all species. These protein sequences were aligned using Muscle 3.7 (Edgar 2004), the ambiguously aligned regions in each alignment were removed using trimAl 1.3 (Capella-Gutierrez et al. 2009) with the nogaps option, and the protein alignments were concatenated using Phyutility 2.2.6 (Smith and Dunn 2008). Phylogenetic analysis of this amino acid dataset was carried out using IQ-Tree 1.6.7 (Nguyen et al. 2015) and the GTR20 + R4 model of sequence evolution. Confidence of branch points was estimated by ultrafast bootstrap approximation with 1,000 replicates.

Nucleotide biases and repeat density in the *Chloropicon* genomes were calculated with nucleotide_biases.pl 1.0a from SYNY (https://github.com/PombertLab/SYNY) on the *Chloropicon* unmasked and masked genomes, respectively. Collinear sets of protein coding genes between the *Chloropicon* genomes were inferred with SYNY from the annotated GenBank (.gbff) files. Normalized sequencing depths were calculated with Coverage_to_Circos.pl 0.2 (this study) from the per nucleotide sequencing depth files generated by get_SNPs.pl 2.0f from the output of samtools's depth -aa function on the *Chloropicon* unmasked genomes. These together with Circos configuration and data files generated with SYNY (gap = 0) were curated manually and plotted with Circos 0.69 to 9 (Krzywinski et al. 2009).

Average nucleotide identity (ANI) metrics were inferred with FastANI 1.34 (Jain et al. 2018) in batch mode with run_fastANI.pl 0.2 (this study) whereas average sequence identity between genomes were inferred from minimap2 2.26 (Li 2021) alignments with paf_metrics.py 0.2c from SYNY.

### Dataset and Assembly Metrics

Sequencing and assembly results were aggregated with MultiQC 1.19 (Ewels et al. 2016) (File S4). Long read data metrics in the MultiQC report were summarized (in.json format) and plotted with keep_longest_reads.pl 0.9b and read_len_plot.py 0.5f, respectively (https://github.com/PombertLab/Misc). Assembly metrics were summarized with QUAST 5.2 (Mikheenko et al. 2018).

## Supplementary Material

evaf140_Supplementary_Data

## Data Availability

The *Chloropicon* genomes were deposited in NCBI under the following accession numbers: *C. primus* RCC138 (BioProject: PRJNA632906; BioSample: SAMN14927950; Accessions: CP060754 to CP060773), *C. roscoffensis* RCC2335 (BioProject: PRJNA596499; BioSample: SAMN13625102; Accessions: CP145460 to CP145478), *C. roscoffensis* RCC1871, principal haplotype (BioProject: PRJNA629116; BioSample: SAMN14770584; Accessions: CP151501 to CP151519), *C. roscoffensis* RCC1871, alternate haplotype (BioProject: PRJNA1081905; BioSample: SAMN14770584; Accessions: CP151482 to CP151500), and *C. roscoffensis* CCMP1998 (BioProject: PRJNA634083; BioSample: SAMN14981274; Accessions: CP145441 to CP145459). Sequencing data were deposited in the NCBI Sequence Read Archive (Table S2). All custom scripts written as part of this study are freely available on GitHub (https://github.com/PombertLab) in the Publication_scripts/2025_Chloropicon_MS_scripts subdirectory.
