# Supplementary material for: Comparative Genomics of Chloropicon primus and Chloropicon roscoffensis Provide Insights into the Evolutionary Dynamics and Ecological Success of These Tiny Green Algae in Marine Environments
Source: Genome Biol Evol. 2025 Jul 11;17(7):evaf140. doi: 10.1093/gbe/evaf140 (PMC12301720; doi:10.1093/gbe/evaf140)
Supplement: evaf140_Supplementary_Data [file evaf140_supplementary_data.zip › Supplementary_tables&figures.pdf]

Supplementary Table S1. Telomere to Telomere (T2T) status of *C. primus* and *C. roscoffensis* chromosomes.

	CCMP1205		RCC138		RCC2335		CCMP1998		RCC1871	
**Chromosome**	**Size (bp)**	**T2T status**	**Size (bp)**	**T2T status**	**Size (bp)**	**T2T status**	**Size (bp)**	**T2T status**	**Size (bp)**	**T2T status**
**1**	1,876,603	complete	1,855,637	partial, 5' telomere missing	2,040,242	complete	2,046,996	complete	1,787,833	partial, 3' telomere missing
**2**	1,764,478	complete	1,769,006	complete	1,605,578	complete	1,618,104	complete	1,587,267	partial, 3' telomere missing
**3**	1,475,055	complete	1,503,776	complete	1,259,922	complete	1,404,726	complete	1,544,382	complete
**4**	1,467,028	complete	1,460,551	complete	1,126,308	complete	1,262,971	complete	1,247,473	complete
**5**	1,105,792	complete	1,118,318	complete	1,064,001	complete	1,144,364	complete	1,156,015	complete
**6**	1,089,399	complete	1,154,373	complete	1,055,140	complete	1,112,205	complete	1,128,824	complete
**7**	832,080	complete	832,941	complete	983,956	complete	949,145	complete	958,571	complete
**8**	802,619	complete	860,321	complete	951,449	complete	887,790	complete	917,703	partial, 5' telomere missing
**9**	781,388	complete	767,277	complete	906,683	complete	830,122	complete	830,532	complete
**10**	755,390	complete	770,552	complete	828,449	complete	788,984	partial, 3' telomere missing	798,944	complete
**11**	725,311	complete	730,620	complete	796,235	complete	705,347	complete	701,900	complete
**12**	633,266	complete	624,486	complete	729,933	complete	640,363	complete	651,014	complete
**13**	602,165	complete	605,067	complete	629,370	complete	627,770	complete	640,473	partial, 3' telomere missing
**14**	585,392	complete	589,408	complete	594,089	complete	594,074	complete	600,075	complete
**15**	580,184	complete	575,298	complete	547,390	complete	580,429	complete	584,536	complete
**16**	567,218	complete	561,312	complete	512,335	complete	510,664	complete	517,919	complete
**17**	498,022	complete	479,965	complete	420,967	complete	419,160	complete	430,955	complete
**18**	477,077	complete	478,129	complete	412,905	complete	382,029	complete	368,489	partial, 5' telomere missing
**19**	413,305	complete	429,915	complete	326,295	complete	327,266	complete	367,329	partial, 5' and 3' telomeres missing
**20**	368,919	complete	417,210	complete						

Supplementary Table S2. *Chloropicon* NCBI SRA data.

**Species**	**Source**	**BioProject**	**Biosample**	**SRA accessions**	**Title**	**No. of bases**
C. roscoffensis RCC1871	This study	PRJNA629116	SAMN14770584	SRR27893522	RNAseq Illumina HiSeq 4000 sequencing of Chloropicon roscoffensis RCC1871 cDNAs	44.2G
	This study	PRJNA629116	SAMN14770584	SRR27893523	Random PacBio Sequel sequencing of Chloropicon roscoffensis RCC1871 genome (SMRT_2)	2.1G
	This study	PRJNA629116	SAMN14770584	SRR27893526	Random Illumina MiSeq sequencing of Chloropicon roscoffensis RCC1871 genome (run_1)	2.7G
	This study	PRJNA629116	SAMN14770584	SRR27893525	Random Illumina MiSeq sequencing of Chloropicon roscoffensis RCC1871 genome (run_2)	1.1G
	This study	PRJNA629116	SAMN14770584	SRR27893524	Random PacBio Sequel sequencing of Chloropicon roscoffensis RCC1871 genome (SMRT_1)	2.2G
C. roscoffensis CCMP1998	This study	PRJNA634083	SAMN14981274	SRR27907040	Random Illumina HiSeq 4000 sequencing of Chloropicon roscoffensis CCMP1998 genome	63G
	This study	PRJNA634083	SAMN14981274	SRR27907038	Random PacBio Sequel sequencing of Chloropicon roscoffensis CCMP1998 genome (SMRT_2)	4.7G
	This study	PRJNA634083	SAMN14981274	SRR27907039	Random PacBio Sequel sequencing of Chloropicon roscoffensis CCMP1998 genome (SMRT_1)	5.1G
	Keeling et al. [a]	PRJNA248394	SAMN02740170	SRR1300422	Whole transcriptome sequencing of Pycnococcus CCMP1998 - MMETSP1085	1.8G
C. roscoffensis RCC2335	This study	PRJNA596499	SAMN13625102	SRR27895190	RNAseq Illumina HiSeq 4000 sequencing of Chloropicon roscoffensis RCC2335 cDNAs	47.2G
	This study	PRJNA596499	SAMN13625102	SRR27895192	Random PacBio Sequel sequencing of Chloropicon roscoffensis RCC2335 genome (SMRT_1)	1.3G
	This study	PRJNA596499	SAMN13625102	SRR27895191	Random PacBio Sequel sequencing of Chloropicon roscoffensis RCC2335 genome (SMRT_2)	1.2G
	This study	PRJNA596499	SAMN13625102	SRR27895194	Random Illumina MiSeq sequencing of Chloropicon roscoffensis RCC2335 genome	3.9G
	This study	PRJNA596499	SAMN13625102	SRR27895193	Random Oxford Nanopore sequencing of Chloropicon roscoffensis RCC2335 genome	2.2G
C. primus RCC138	This study	PRJNA632906	SAMN14927950	SRR27893603	Random Illumina MiSeq sequencing of Chloropicon roscoffensis RCC138 genome (run_2)	1.4G
	This study	PRJNA632906	SAMN14927950	SRR27893604	Random Illumina MiSeq sequencing of Chloropicon roscoffensis RCC138 genome (run_1)	4.1G
	This study	PRJNA632906	SAMN14927950	SRR27893602	Random Oxford Nanopore sequencing of Chloropicon roscoffensis RCC138 genome	6.4G
C. primus CCMP1205	Lemieux et al. [b]	PRJNA316521	SAMN04584780	SRR8992761	RNAseq of Chloropicon primus	9G
	Lemieux et al. [b]	PRJNA316521	SAMN04584780	SRR8185495	PacBio_RSII_1	950.6M
	Lemieux et al. [b]	PRJNA316521	SAMN04584780	SRR8185494	PacBio_RSII_2	1.1G
	Lemieux et al. [b]	PRJNA316521	SAMN04584780	SRR8185493	PacBio_RSII_3	1.1G
	Lemieux et al. [b]	PRJNA316521	SAMN04584780	SRR8185492	PacBio_RSII_4	723.8M
	Lemieux et al. [b]	PRJNA316521	SAMN04584780	SRR8185496	Mate_pair illumina	3.6G
	Lemieux et al. [b]	PRJNA316521	SAMN04584780	SRR8185497	Paired_end illumina	4.5G

[a] Keeling PJ et al. **The Marine Microbial Eukaryote Transcriptome Sequencing Project (MMETSP): illuminating the functional diversity of eukaryotic life in the oceans through transcriptome sequencing.** PLoS Biol. 2014 Jun 24;12(6):e1001889. doi: 10.1371/journal.pbio.1001889. PMID: 24959919; PMCID: PMC4068987.

[b] Lemieux C, Turmel M, Otis C, Pombert JF. **A streamlined and predominantly diploid genome in the tiny marine green alga *Chloropicon primus*.** Nat Commun. 2019 Sep 6;10(1):4061. doi: 10.1038/s41467-019-12014-x. PMID: 31492891; PMCID: PMC673126

Supplementary Table S3. Filtration statistics of NCBI SRA data.

**Species**	**SRA Accessions**	**Title**	**No. of reads**	**No. of bases**	**No. of filtered reads**	**No. of filtered bases**
C. roscoffensis RCC1871	SRR27893522	RNAseq Illumina HiSeq 4000 sequencing of Chloropicon roscoffensis RCC1871 cDNAs	147.2M	44.2G	143.2M	41.1G
	SRR27893523	Random PacBio Sequel sequencing of Chloropicon roscoffensis RCC1871 genome (SMRT_2)	449K	2.1G	423K	2.0G
	SRR27893526	Random Illumina MiSeq sequencing of Chloropicon roscoffensis RCC1871 genome (run_1)	5.3M	2.7G	4.8M	2.5G
	SRR27893525	Random Illumina MiSeq sequencing of Chloropicon roscoffensis RCC1871 genome (run_2)	2.0M	1.1G	1.9M	1.0G
	SRR27893524	Random PacBio Sequel sequencing of Chloropicon roscoffensis RCC1871 genome (SMRT_1)	474K	2.2G	453K	2.1G
C. roscoffensis CCMP1998	SRR27907040	Random Illumina HiSeq 4000 sequencing of Chloropicon roscoffensis CCMP1998 genome	210M	63G	199M	58.7G
	SRR27907038	Random PacBio Sequel sequencing of Chloropicon roscoffensis CCMP1998 genome (SMRT_2)	672K	4.7G	327K	4.4G
	SRR27907039	Random PacBio Sequel sequencing of Chloropicon roscoffensis CCMP1998 genome (SMRT_1)	721K	5.1G	669K	4.8G
C. roscoffensis RCC2335	SRR27895190	RNAseq Illumina HiSeq 4000 sequencing of Chloropicon roscoffensis RCC2335 cDNAs	157M	47.2G	153M	44.0G
	SRR27895192	Random PacBio Sequel sequencing of Chloropicon roscoffensis RCC2335 genome (SMRT_1)	387K	1.3G	374K	1.3G
	SRR27895191	Random PacBio Sequel sequencing of Chloropicon roscoffensis RCC2335 genome (SMRT_2)	418K	1.2G	400K	1.2G
	SRR27895194	Random Illumina MiSeq sequencing of Chloropicon roscoffensis RCC2335 genome	7.4M	3.9G	6.7M	3.6G
	SRR27895193	Random Oxford Nanopore sequencing of Chloropicon roscoffensis RCC2335 genome	1.3M	2.2G	684K	1.9G
C. primus RCC138	SRR27893603	Random Illumina MiSeq sequencing of Chloropicon roscoffensis RCC138 genome (run_2)	2.6M	1.4G	2.4M	1.3G
	SRR27893604	Random Illumina MiSeq sequencing of Chloropicon roscoffensis RCC138 genome (run_1)	8.1M	4.1G	7.4M	3.8G
	SRR27893602	Random Oxford Nanopore sequencing of Chloropicon roscoffensis RCC138 genome	1.5M	6.4G	1.0M	6.2G

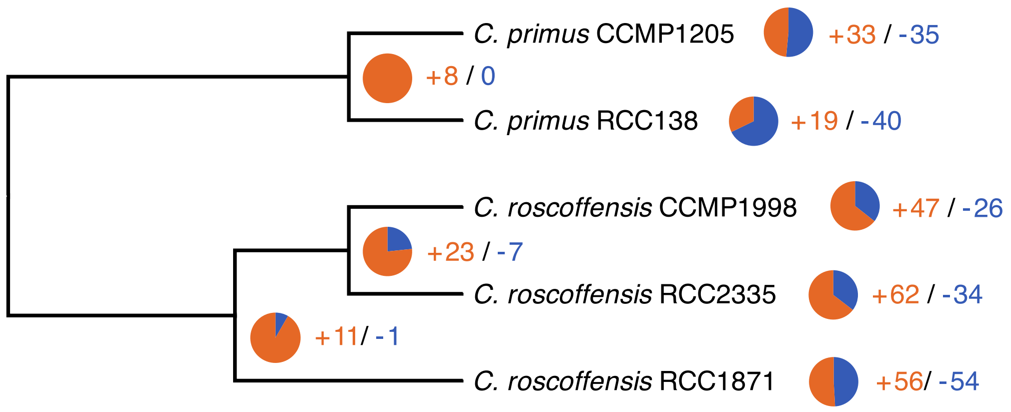

Supplementary figure S1. Gene family expansion and contraction patterns among *Chloropicon* lineages, as estimated by CAFE using the orthogroups identified by OrthoFinder. The numbers next to the pie charts indicate the numbers of expanded (orange) and contracted (blue) orthogroups.

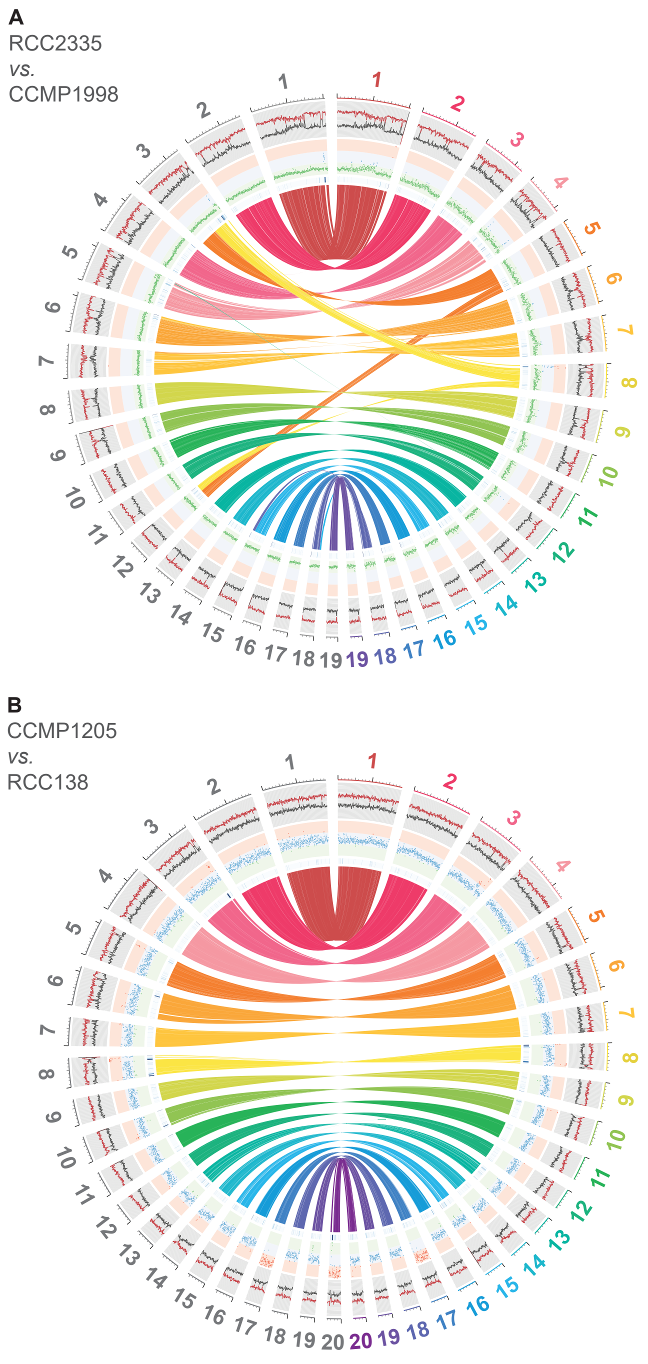

Supplementary figure S2. Chromosomal reorganizations observed between *C. roscoffensis* RCC2335/CCMP1998 **(A)** and between *C. primus* CCMP1205/RCC138 **(B)**. These comparisons were carried out with SYNY. GC and AT percentages are plotted in red and grey, respectively, in the outermost concentric circles (sliding window 10,000 nt, step 5,000 nt). Normalized sequencing depths (Illumina) are plotted in the innermost concentric circles, with the haploid, diploid, and triploid depths being color coded in green, blue, and red, respectively (sliding window 10,000 nt, step 5,000 nt). Colinear blocks are highlighted by color-coded ribbons extending from the reference chromosomes (Panel A, RCC2335; Panel B, CCMP1205) to those from the genomes being compared.

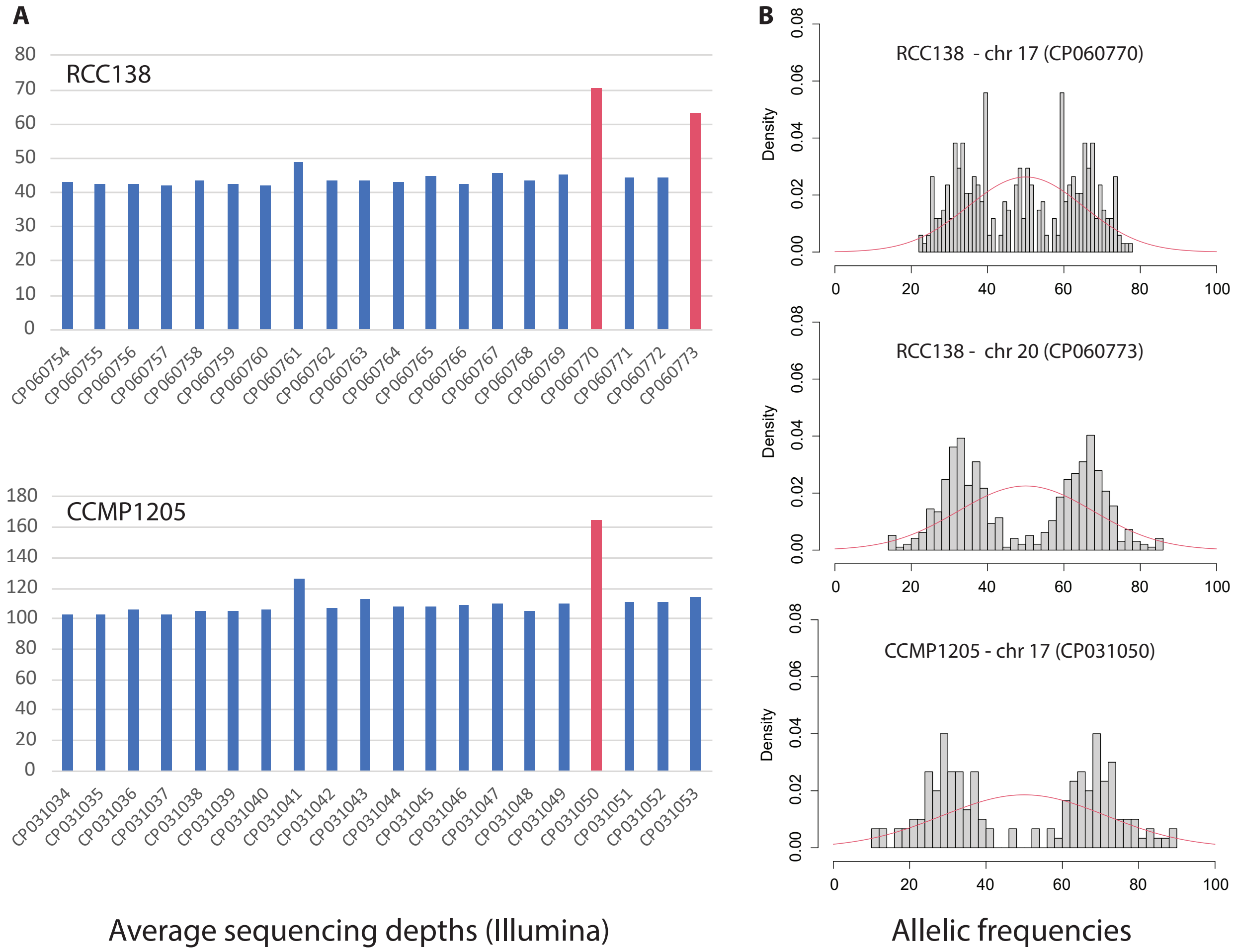

Supplementary figure S3. Aneuploid chromosomes in the *C. primus* RCC138 and CCMP1205 genomes. **A.** Average Illumina sequencing depths per chromosomes. Chromosomes with depths greater than 1.4 times the average are highlighted in magenta. **B.** Allelic frequencies observed for SNPs and indels in the corresponding chromosomes based on read mapping/variant calling analyses with minimap2 and VarScan2.

Gamma carbonic anhydrases (γCAs)

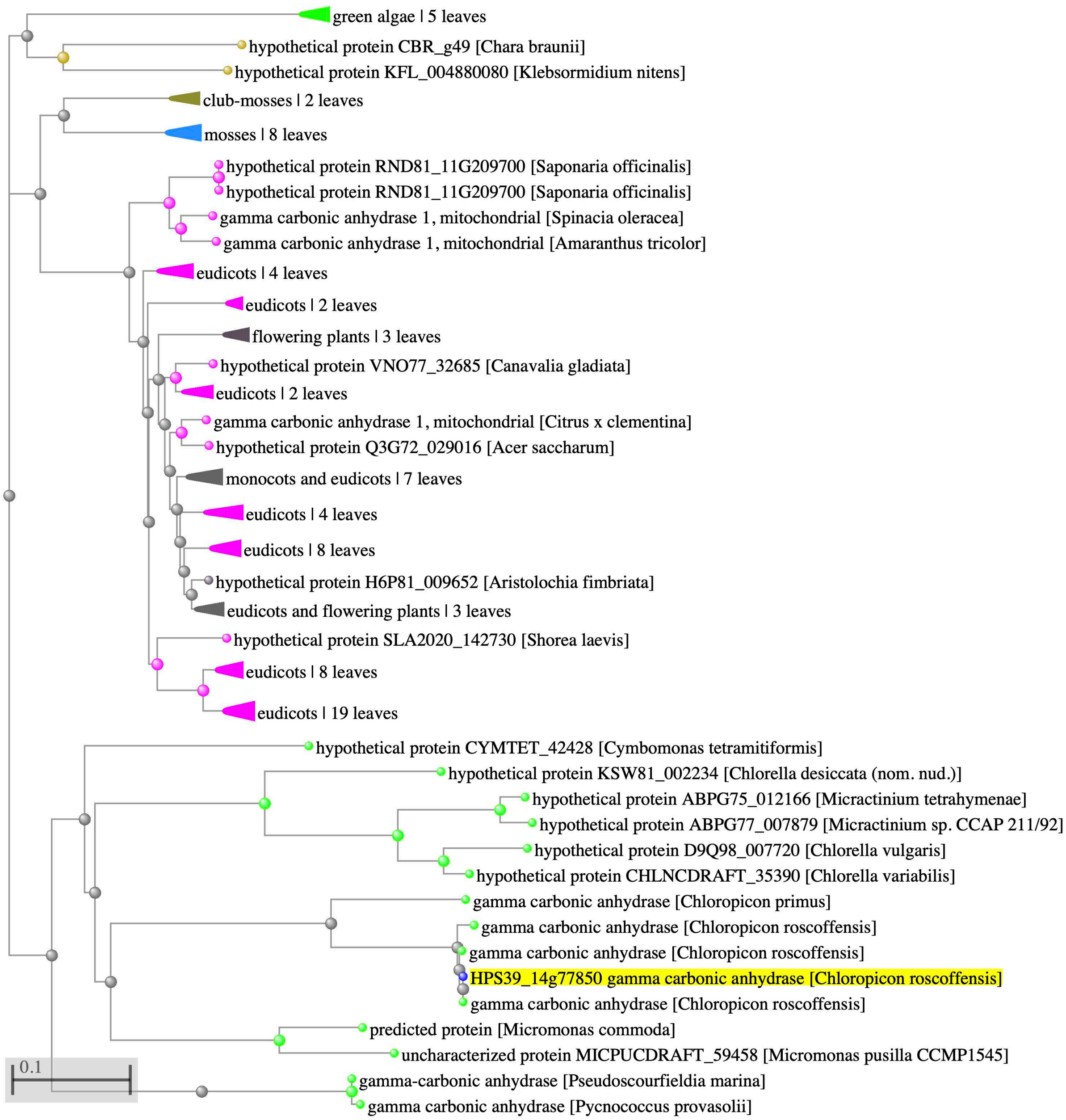

Supplementary figure S4. Gamma carbonic anhydrase. This NJ tree includes the best hits recovered by BlastP searches against the NCBI nr database restricted to the Viridiplantae.

Catalytic subunit QTRT1 of eukaryotic type tRNA-guanine transglycosylases (eTGTs)

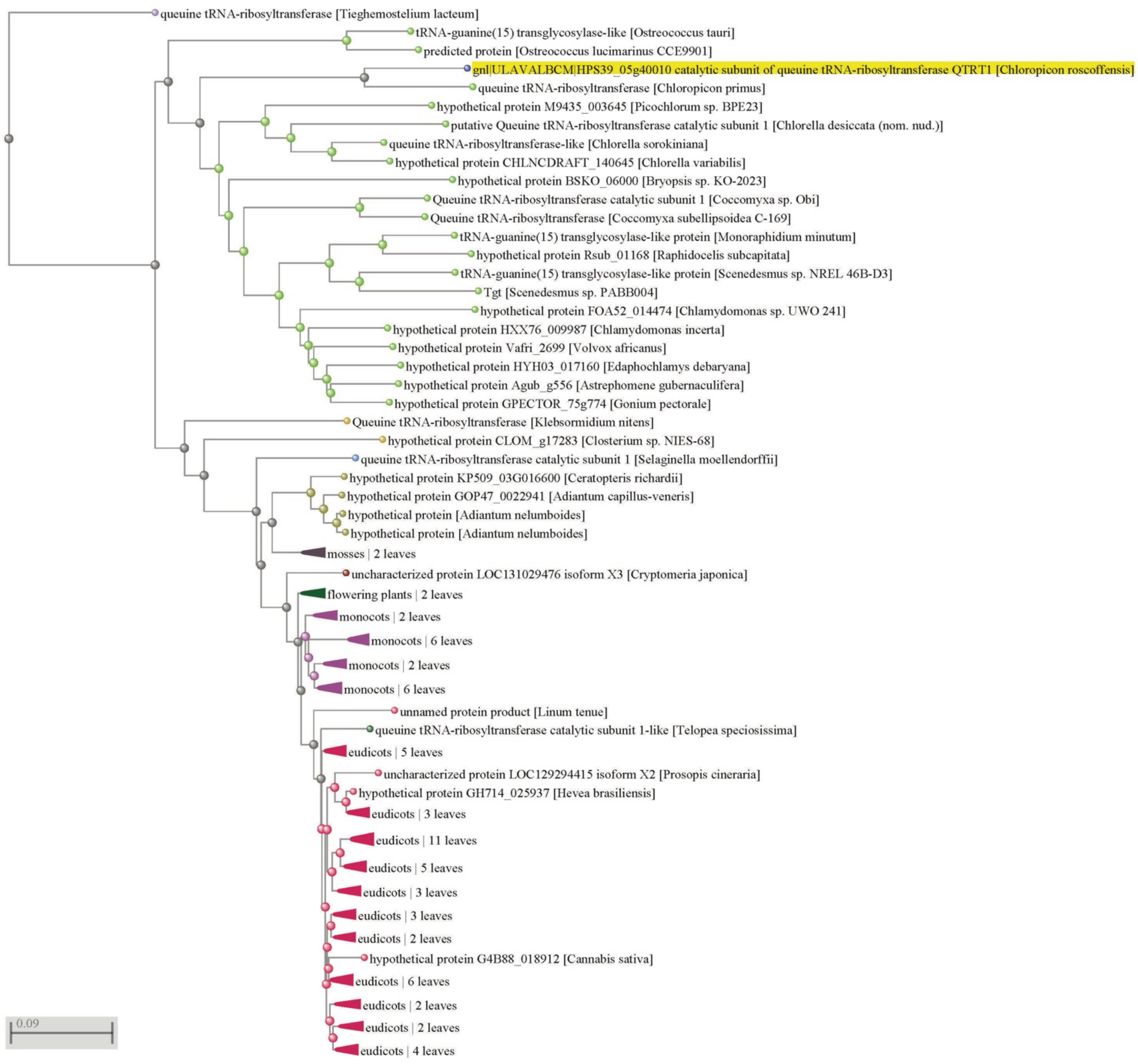

Supplementary figure S5a. Catalytic subunit QTRT1 of eukaryotic type tRNA-guanine transglycosylases (eTGTs). This NJ tree includes the best hits recovered by BlastP searches against the NCBI nr database.

Accessory subunit QTRT2 of eukaryotic type tRNA-guanine transglycosylases (eTGTs)

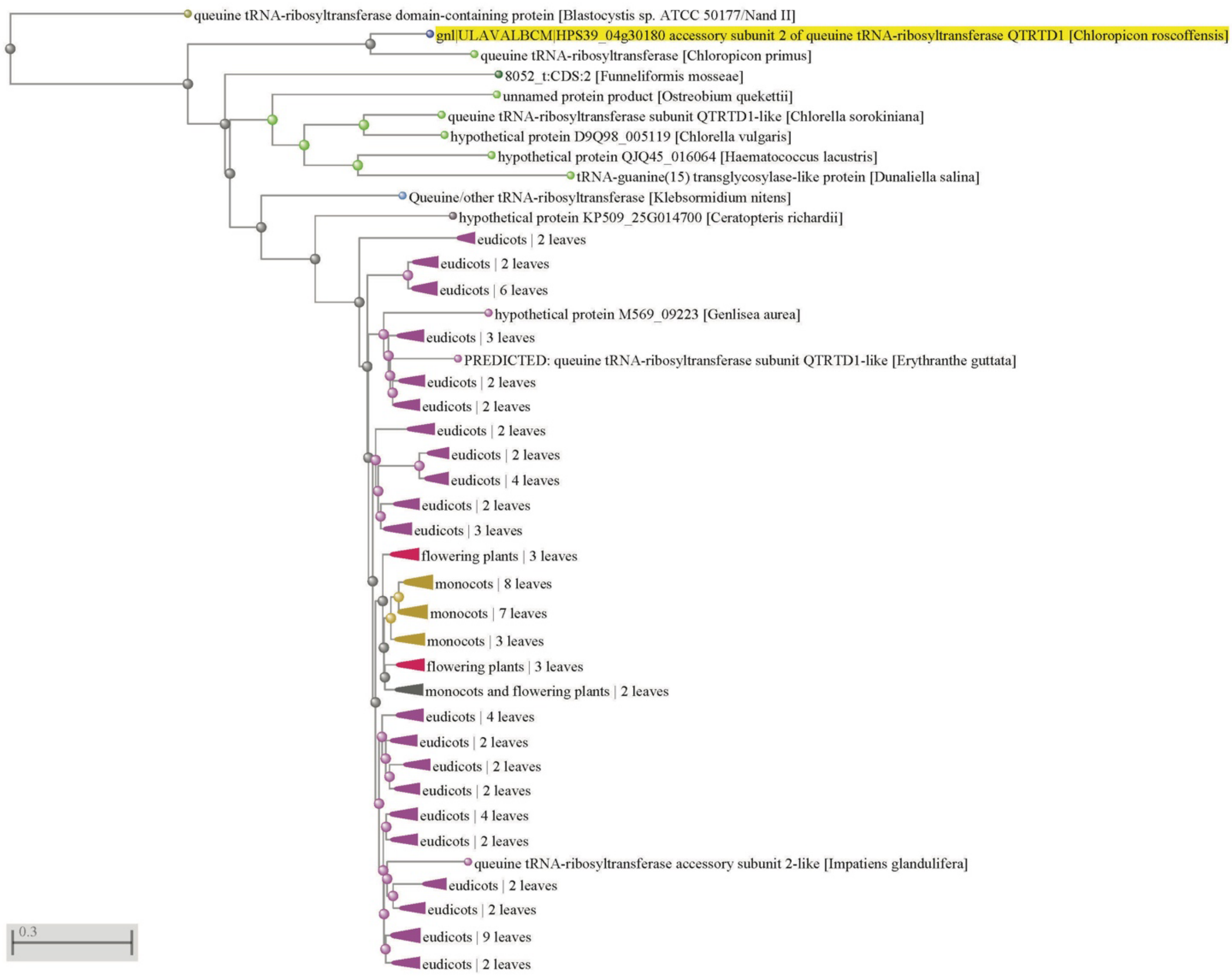

Supplementary figure S5b. Accessory subunit QTRT2 of eukaryotic type tRNA-guanine transglycosylases (eTGTs). This NJ tree includes the best hits recovered by BlastP searches against the NCBI nr database.

Queuosine salvage protein QNG1

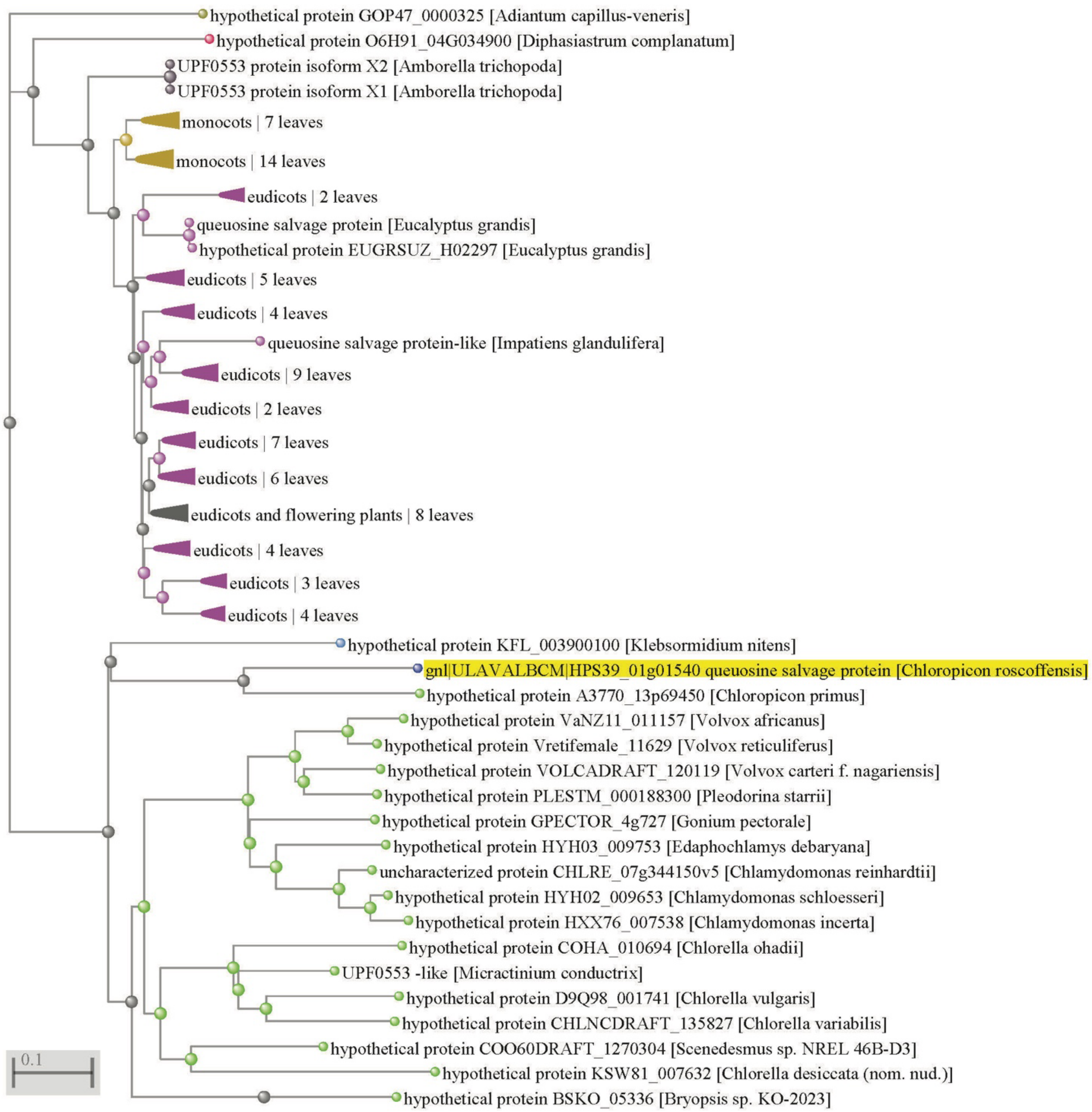

Supplementary figure S5c. Queuosine salvage protein QNG1. This NJ tree includes the best hits recovered by BlastP searches against the NCBI nr database.

Queuosine precursor transporter YhhQ

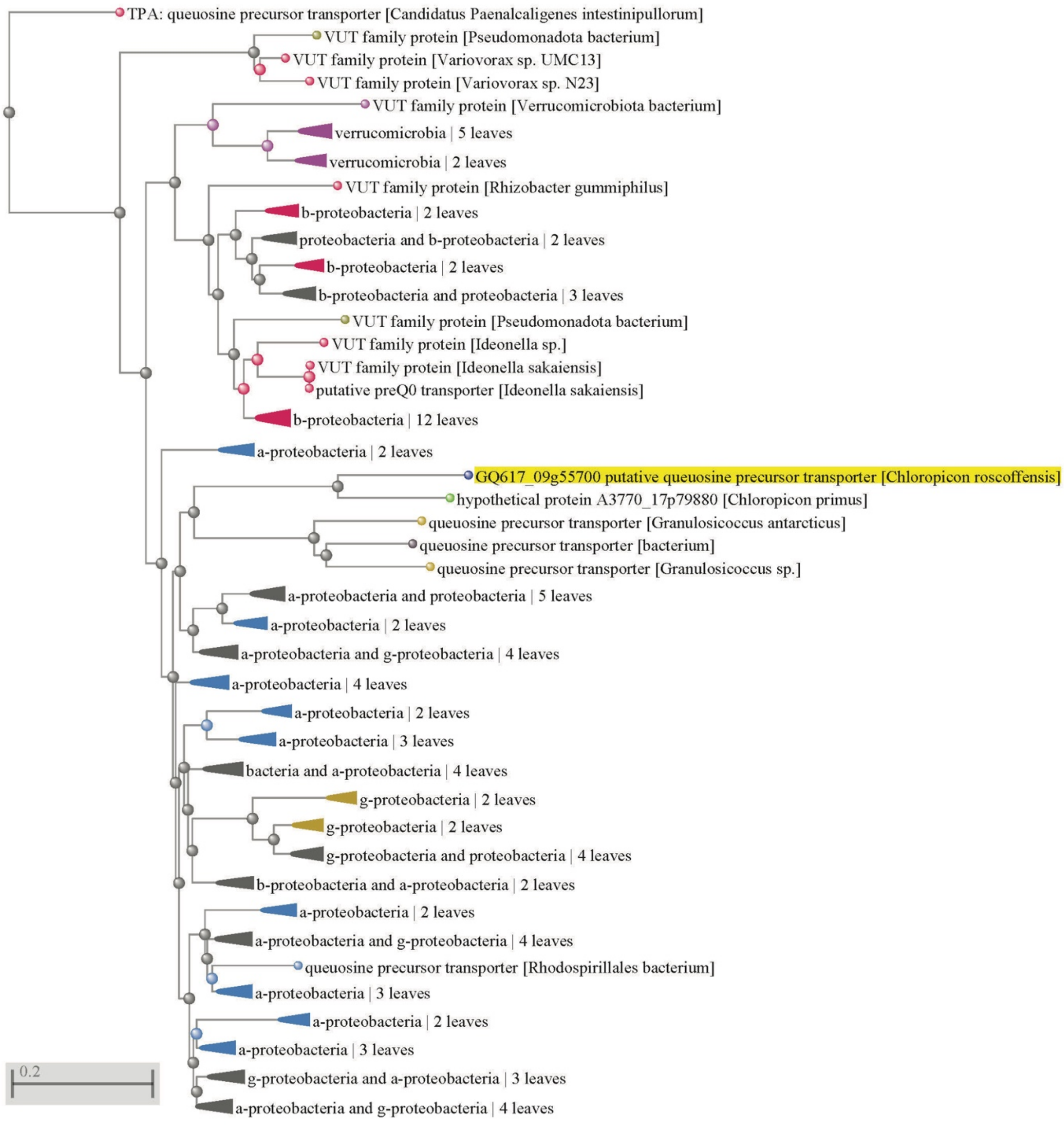

Supplementary figure S5d. Queuosine precursor transporter YhhQ. This NJ tree includes the best hits recovered by BlastP searches against the NCBI nr database.

Bacterial-type tRNA-guanine transglycosylases (BL-TGTs)

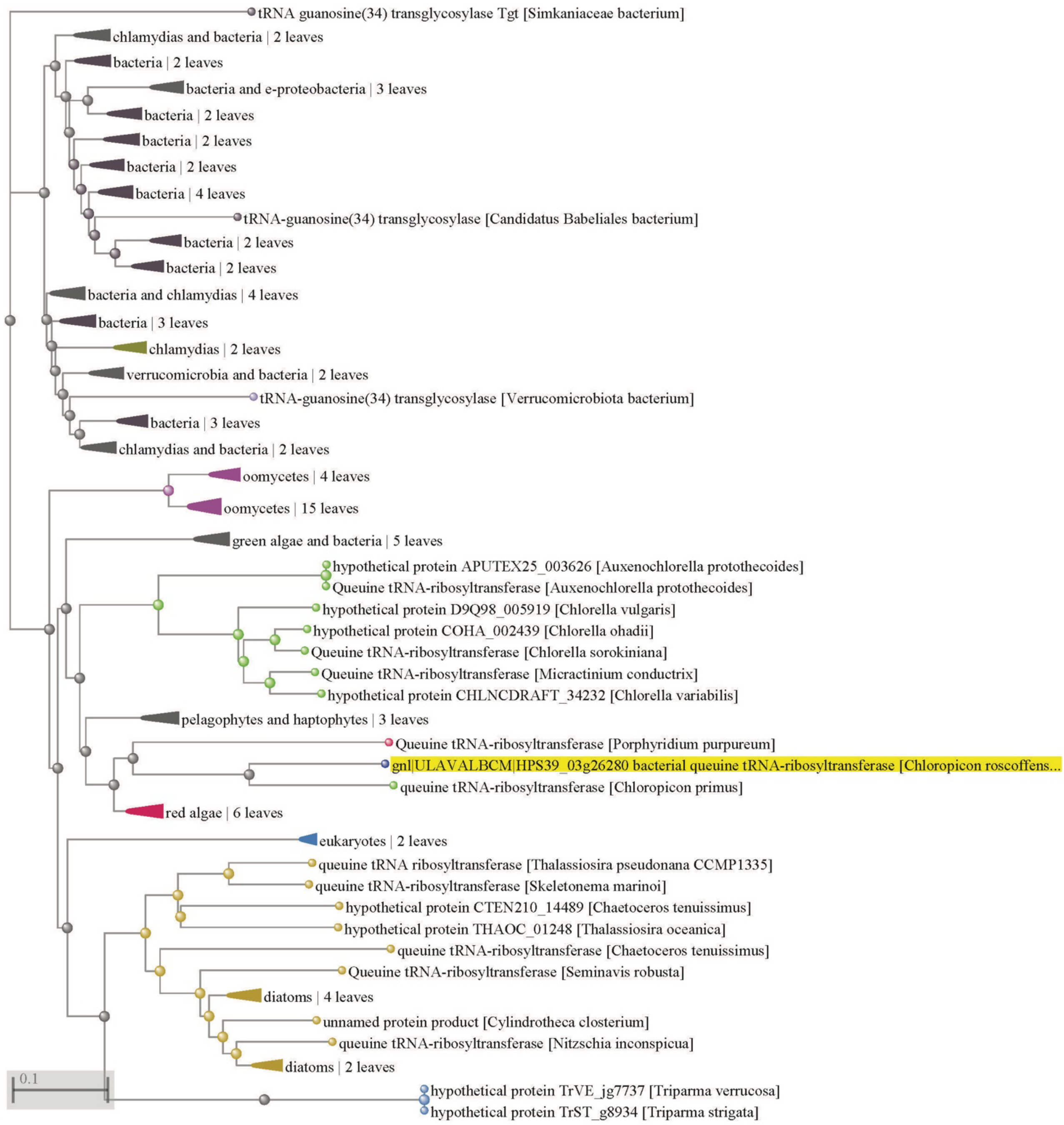

Supplementary figure S5e. Bacterial-type tRNA-guanine transglycosylases (BL-TGTs). This NJ tree includes the best hits recovered by BlastP searches against the NCBI nr database.

QueA tRNA preQ1(34) S-adenosylmethionine ribosyltransferase-isomerase

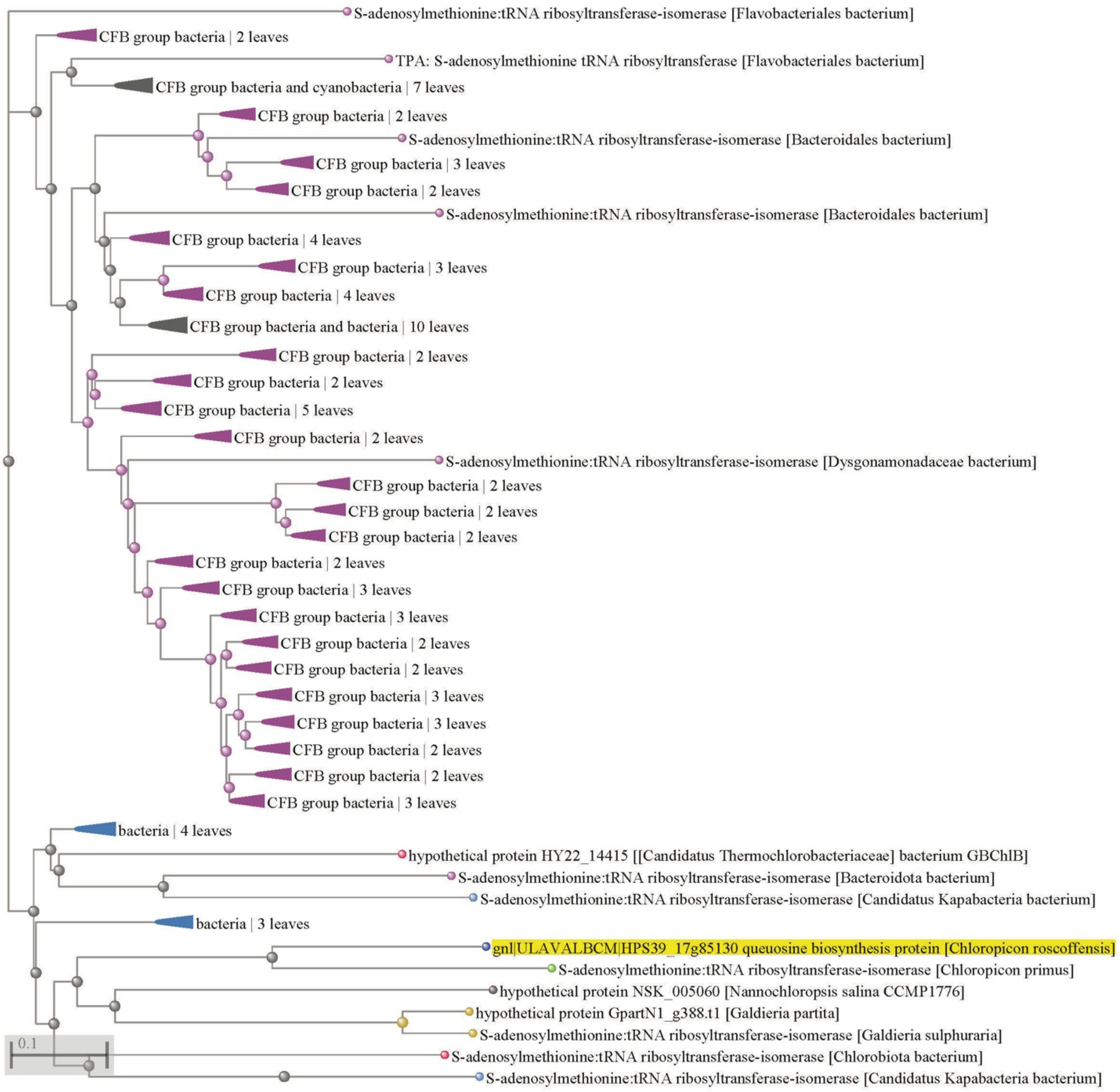

Supplementary figure S5f. QueA (tRNA preQ1(34) S-adenosylmethionine ribosyltransferase-isomerase. This NJ tree includes the best hits recovered by BlastP searches against the NCBI nr database.

QueG tRNA epoxyqueuosine(34) reductase

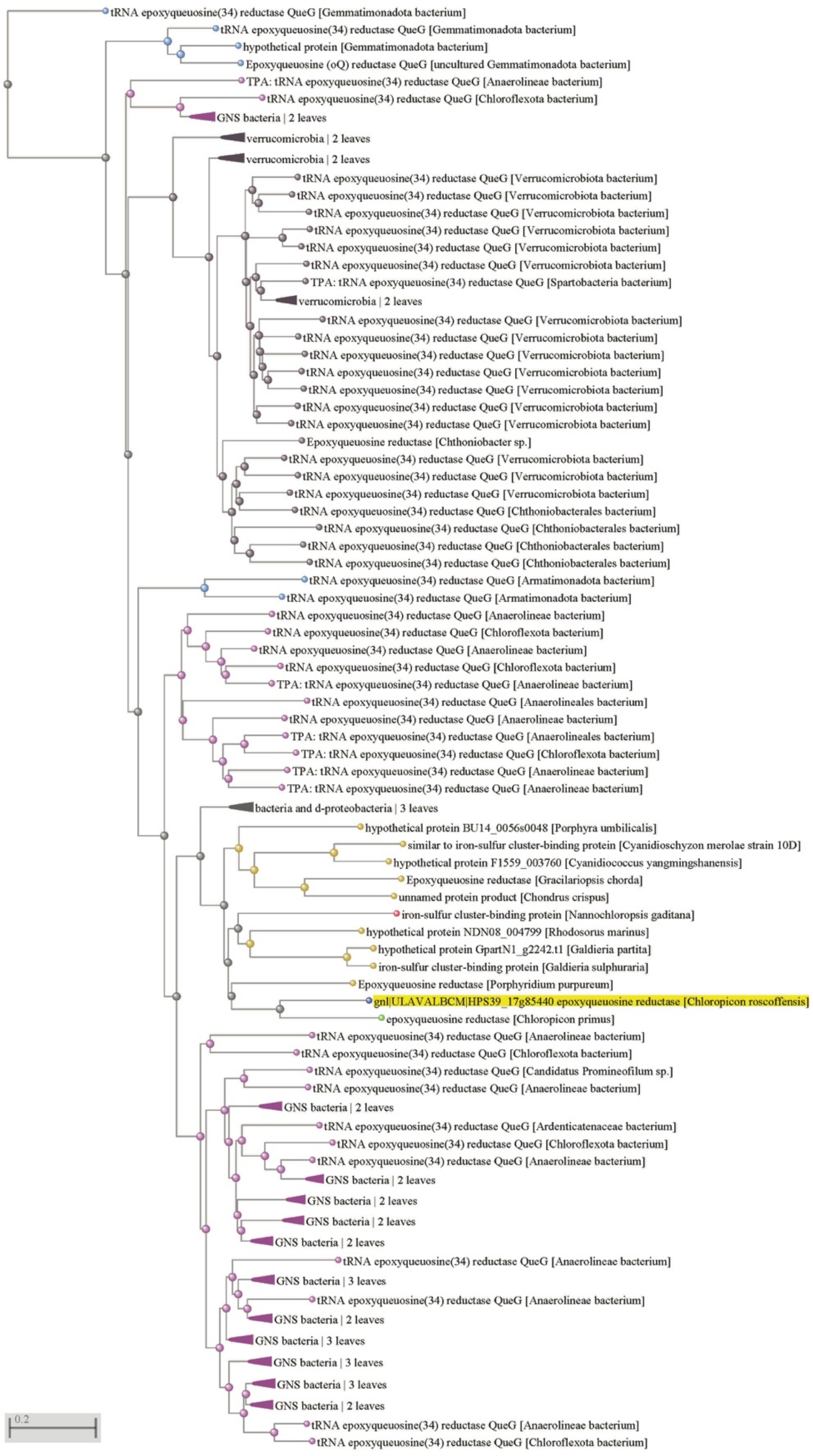

Supplementary figure S5g. QueG tRNA epoxyqueuosine(34) reductase. This NJ tree includes the best hits recovered by BlastP searches against the NCBI nr database.

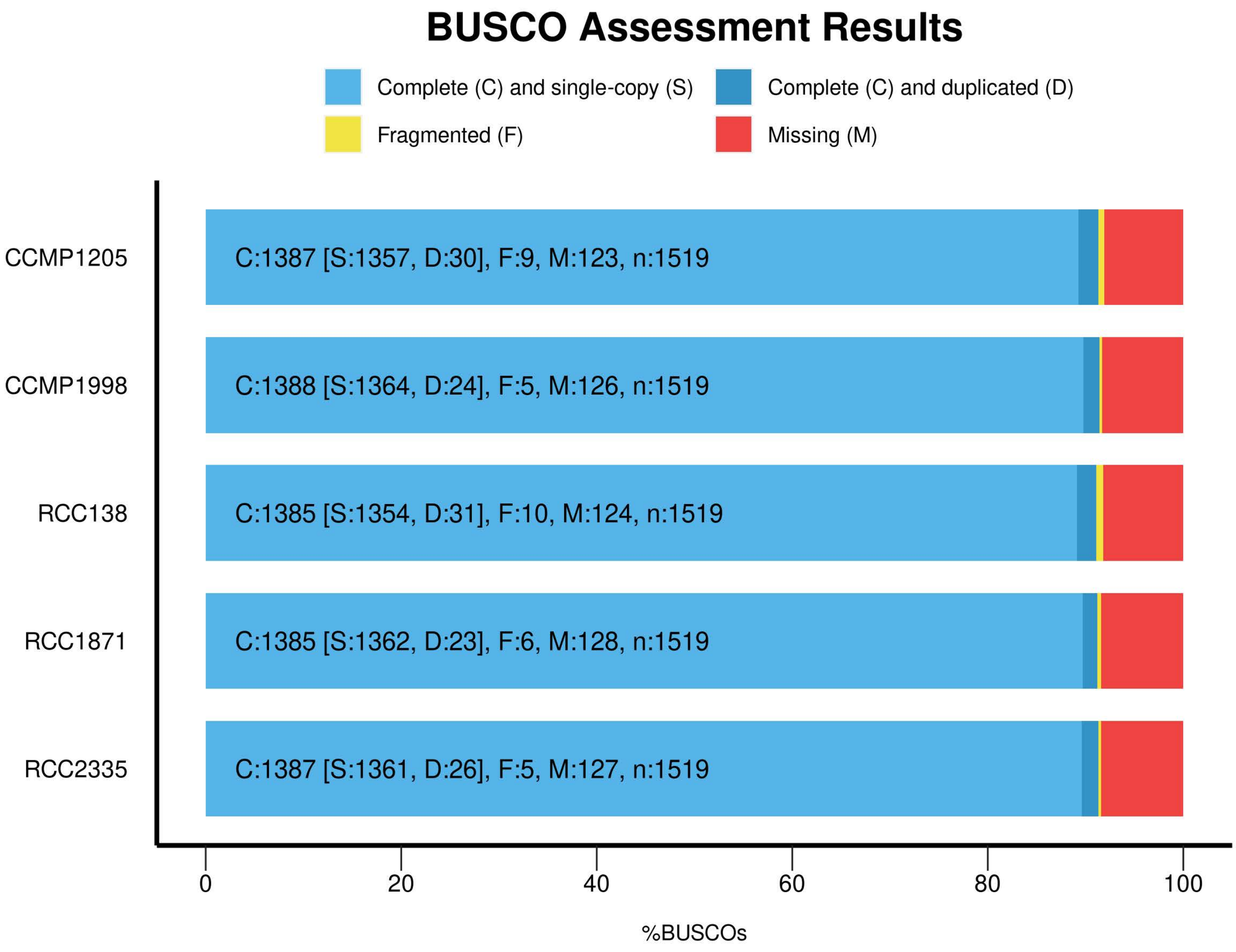

Supplementary figure S6. BUSCO assessment results..
