# Supplementary material for: Comparative Genomics of Chloropicon primus and Chloropicon roscoffensis Provide Insights into the Evolutionary Dynamics and Ecological Success of These Tiny Green Algae in Marine Environments
Source: Genome Biol Evol. 2025 Jul 11;17(7):evaf140. doi: 10.1093/gbe/evaf140 (PMC12301720; doi:10.1093/gbe/evaf140)
Supplement: evaf140_Supplementary_Data [file evaf140_supplementary_data.zip › Supplementary_file_S4.pdf]

multiqc

A modular tool to aggregate results from bioinformatics analyses across many samples into a single report.

Report generated on 2024-02-11, 19:03 EST based on data in: `/mnt/d/LinuxVM/Chloropicon/MultiQC`

General Statistics

Copy table | Configure Columns | Plot | Showing 21/21 rows and 11/15 columns.

Sample Name	N50 (Kbp)	Assembly Length (Mbp)	% Duplication	M Reads After Filtering	GC content	% PF	% Adapter	% Dups	% GC	Median Read Length	M Seqs
**CCMP1205**	1 089.4Kbp	17.4Mbp									
**CCMP1205_R1**			0.4%	8.9	56.2%	46.6%	1.7%	14.3%	54%	247 bp	9.6
**CCMP1205_R2**								16.5%	54%	250 bp	9.6
**CCMP1998**	1 112.2Kbp	16.8Mbp									
**CCMP1998_R1**			3.1%	33.6	59.1%	8.0%	19.4%	48.8%	57%	150 bp	210.1
**CCMP1998_R1.RNAseq**			57.4%	0.0	0.0%	0.0%	1.0%	53.6%	61%	50 bp	18.3
**CCMP1998_R2**								40.9%	57%	150 bp	210.1
**CCMP1998_R2.RNAseq**								49.9%	61%	50 bp	18.3
**RCC138**	1 118.3Kbp	17.6Mbp									
**RCC138_R1**			0.3%	9.9	50.4%	46.1%	1.7%	12.3%	52%	294 bp	10.7
**RCC138_R2**								10.4%	53%	294 bp	10.7
**RCC1871**	1 128.8Kbp	16.8Mbp									
**RCC1871_R1**			0.3%	5.8	57.8%	39.6%	1.6%	12.9%	58%	294 bp	7.4
**RCC1871_R1.RNAseq**			49.5%	101.3	61.8%	34.4%	3.1%	74.6%	61%	150 bp	147.2
**RCC1871_R2**								10.8%	59%	294 bp	7.4
**RCC1871_R2.RNAseq**								71.6%	61%	150 bp	147.2
**RCC2335**	984.0Kbp	16.8Mbp									
**RCC2335_R1**			0.3%	6.5	56.2%	43.9%	1.4%	17.3%	57%	294 bp	7.4
**RCC2335_R1.RNAseq**			50.4%	104.5	61.8%	33.2%	3.5%	75.0%	61%	150 bp	157.3
**RCC2335_R2**								15.3%	58%	294 bp	7.4
**RCC2335_R2.RNAseq**								73.2%	61%	150 bp	157.3

Long read data

Metrics calculated with keep_longest_reads.pl (https://github.com/PombertLab).

Copy table | Configure Columns | Plot | Showing 6/6 rows and 9/9 columns.

Sample Name	Number of reads	Total number of bases	Longest read	Shortest read	Average read size	Median read size	N50	N75	N90
CCMP1205.pacb	653 751	3 833 597 866	160 131	2	5 864	3 937	10 348	6 209	3 562
CCMP1998.pacb	1 393 086	9 762 292 811	113 647	50	7 008	5 047	11 530	6 852	3 805
RCC138.nanopo	1 450 964	6 380 433 157	395 573	1	4 397	2 353	8 914	4 302	2 283
RCC1871.pacbi	922 950	4 235 859 849	108 461	50	4 589	2 957	8 119	4 446	2 414
RCC2335.nanop	1 275 368	2 213 565 881	157 406	1	1 736	1 121	2 907	1 620	809
RCC2335.pacbi	805 340	2 561 487 286	117 137	50	3 181	1 842	5 699	2 976	1 589

CCMP1205 pacbio

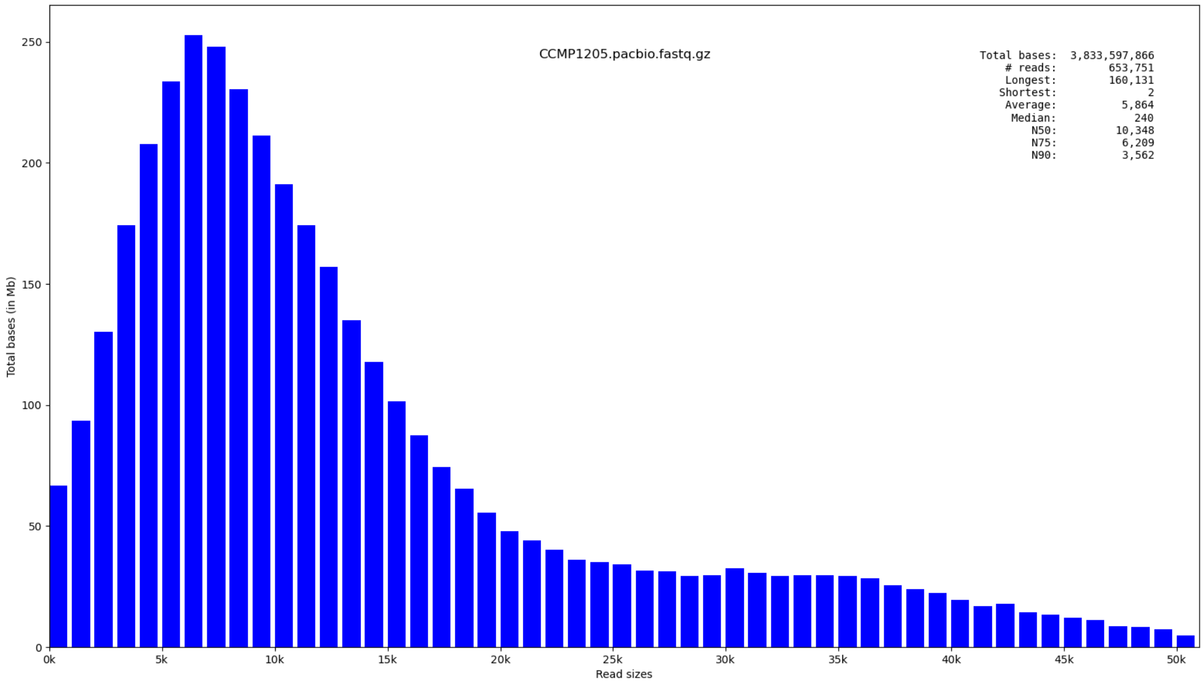

CCMP1998 pacbio

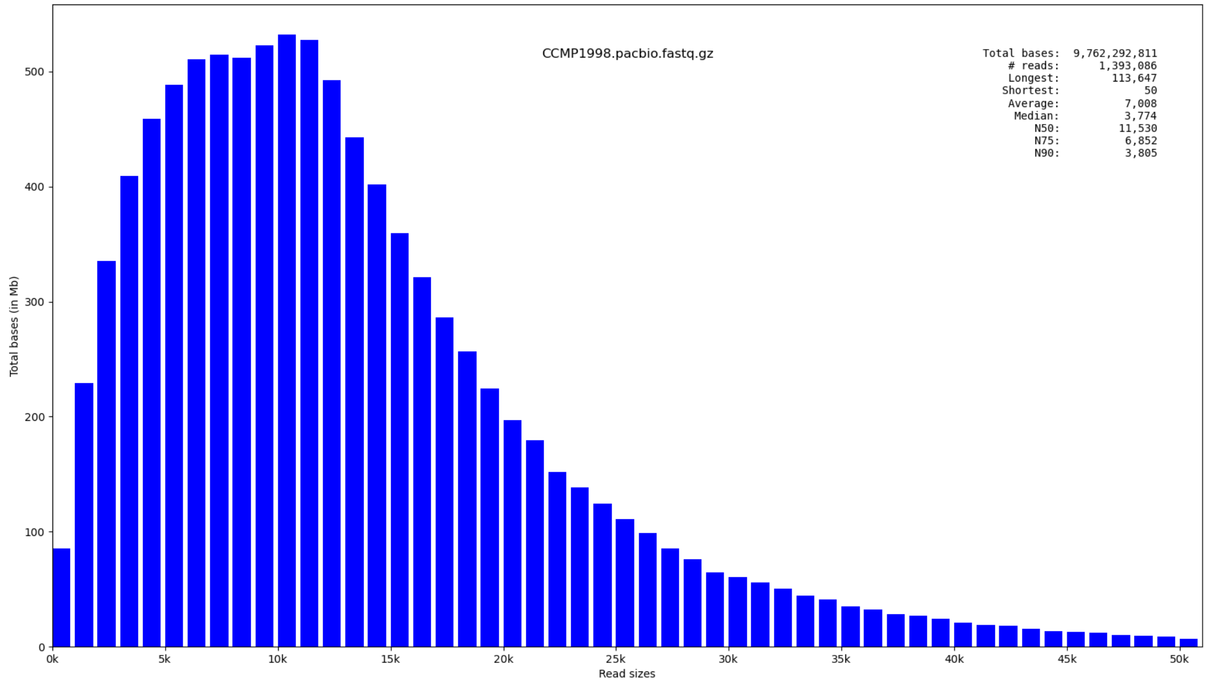

RCC138 nanopore

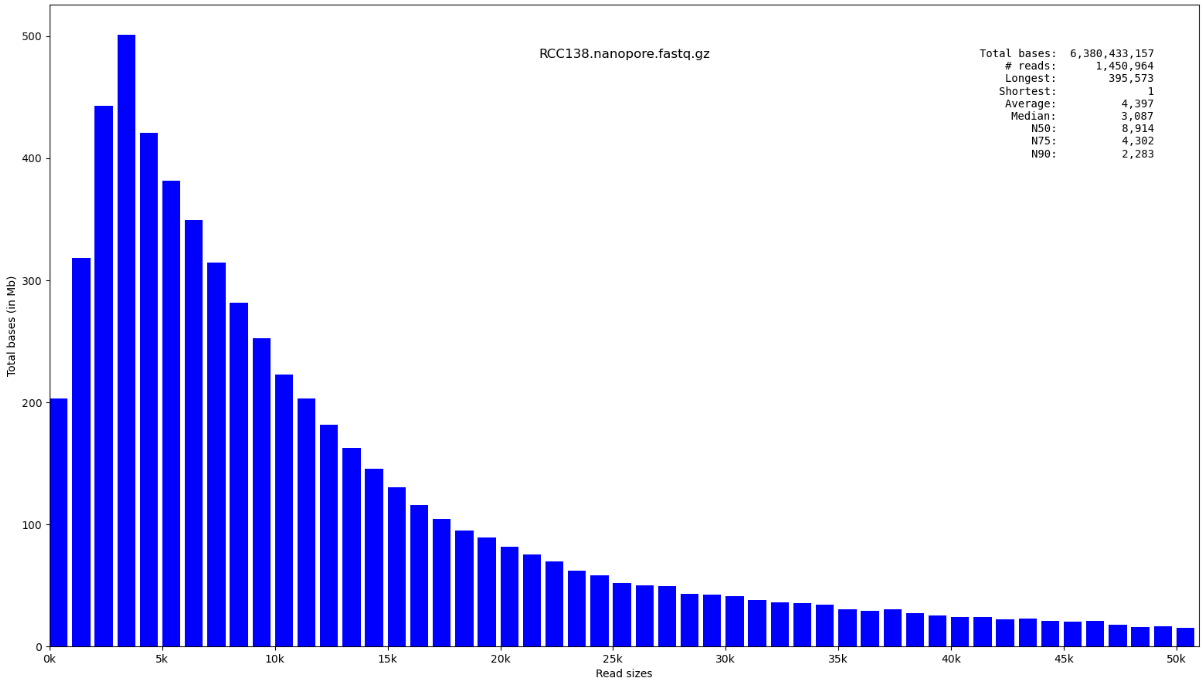

RCC1871 pacbio

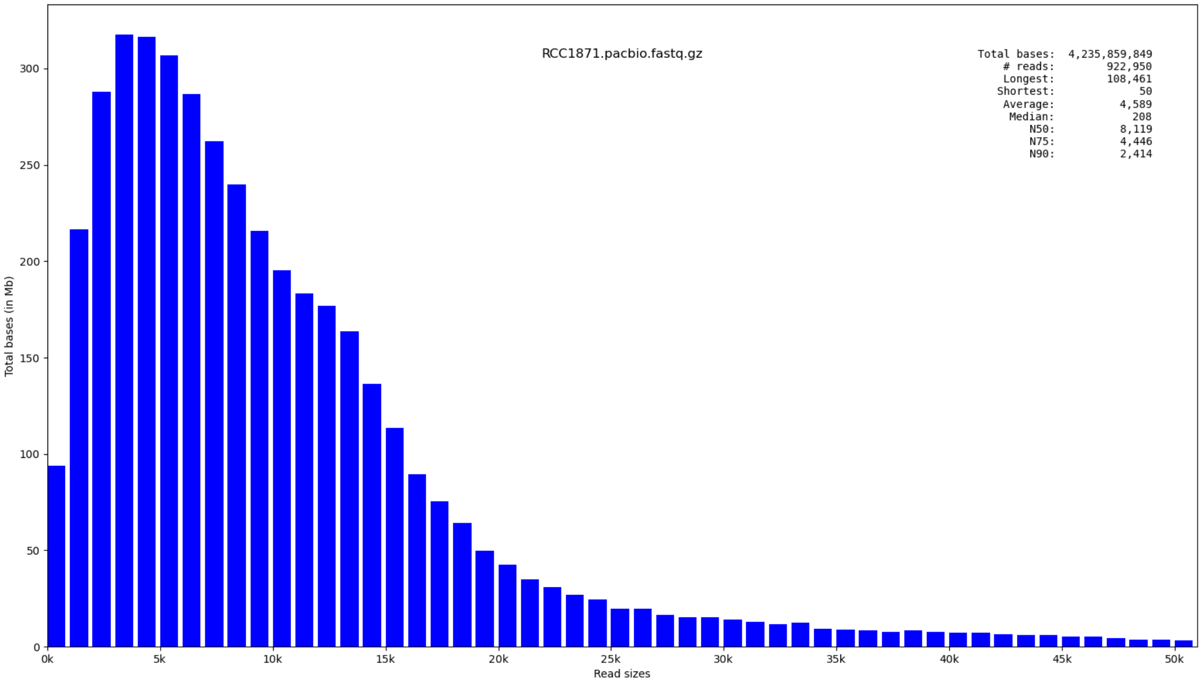

RCC2335 nanopore

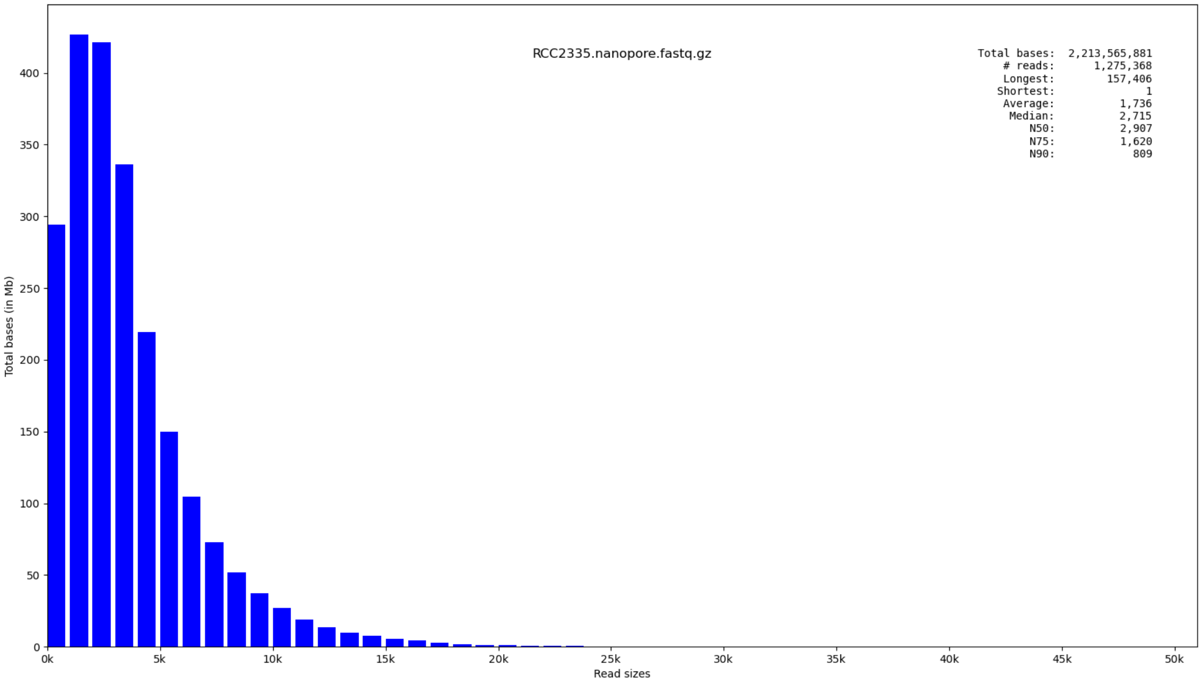

RCC2335 pacbio

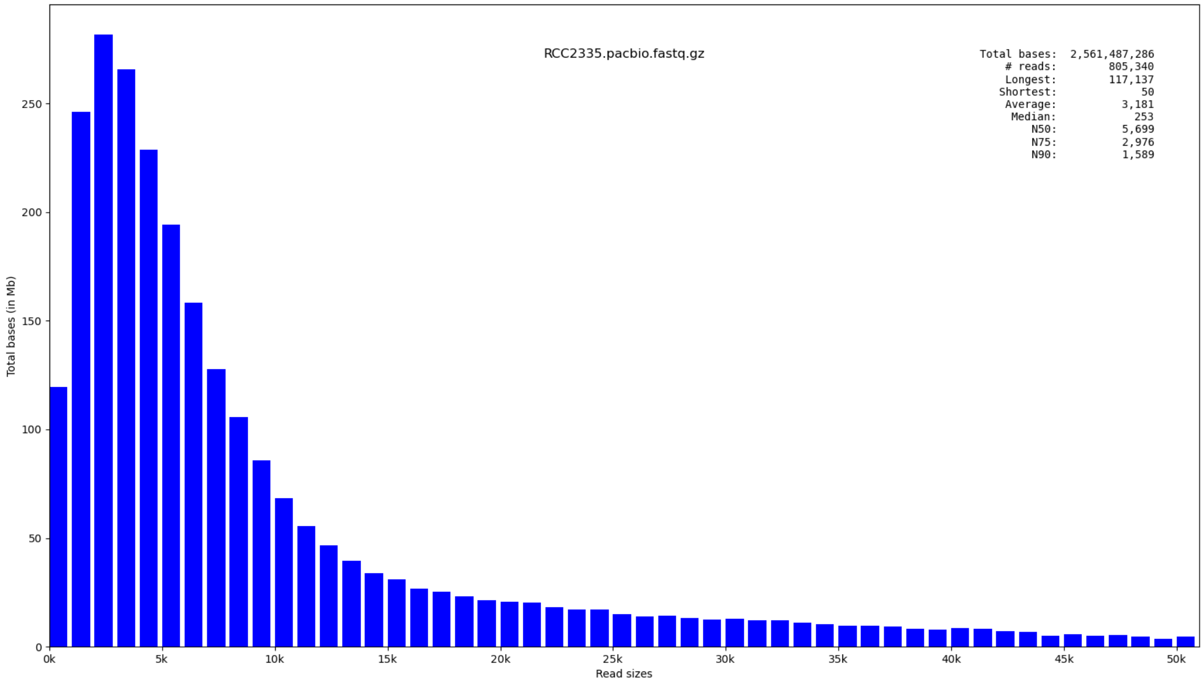

QUAST

QUAST is a quality assessment tool for genome assemblies, written by the Center for Algorithmic Biotechnology. *DOI: 10.1093/bioinformatics/btt086.*

Assembly Statistics

Copy table | Configure Columns | Plot | Showing 5/5 rows and 4/4 columns.

Sample Name	N50 (Kbp)	L50 (K)	Largest contig (Kbp)	Length (Mbp)
**CCMP1205**	1 089.4Kbp	0.0K	1 876.6Kbp	17.4Mbp
**CCMP1998**	1 112.2Kbp	0.0K	2 047.0Kbp	16.8Mbp
**RCC138**	1 118.3Kbp	0.0K	1 855.6Kbp	17.6Mbp
**RCC1871**	1 128.8Kbp	0.0K	1 787.8Kbp	16.8Mbp
**RCC2335**	984.0Kbp	0.0K	2 040.2Kbp	16.8Mbp

Number of Contigs

This plot shows the number of contigs found for each assembly, broken down by length.

Counts | Percentages

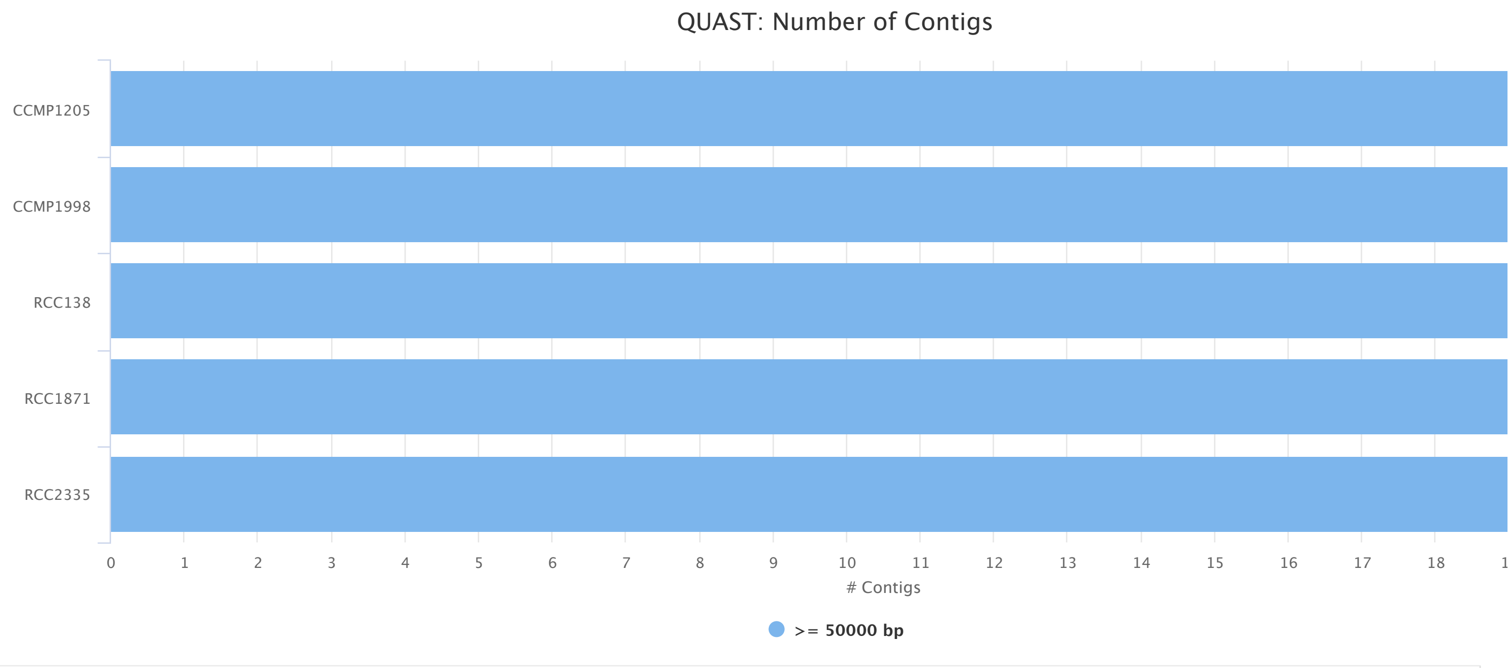

BUSCO *Version:* `5.6.1`

BUSCO assesses genome assembly and annotation completeness with Benchmarking Universal Single-Copy Orthologs. *DOI: 10.1093/bioinformatics/btv351.*

Lineage: chlorophyta_odb10

Number of BUSCOs | Percentages

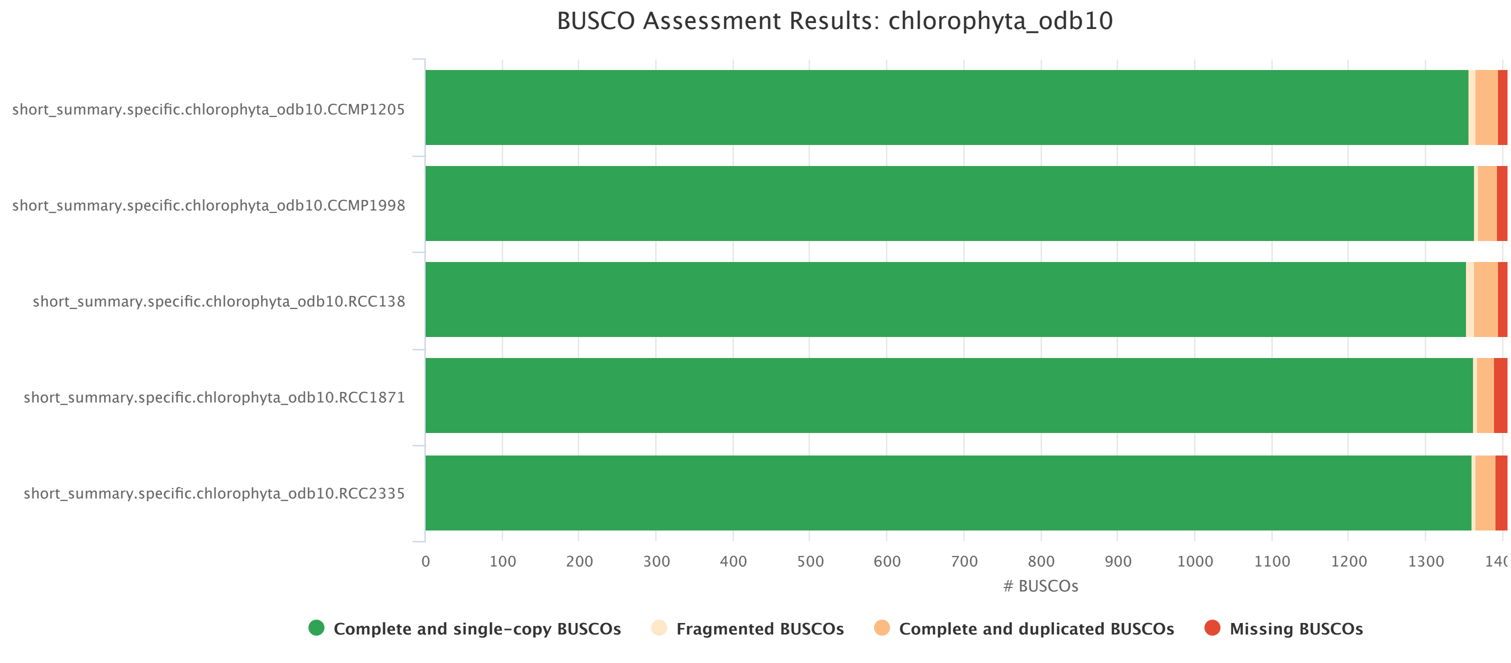

fastp *Version:* `0.23.4`

fastp An ultra-fast all-in-one FASTQ preprocessor (QC, adapters, trimming, filtering, splitting...). *DOI: 10.1093/bioinformatics/bty560.*

Filtered Reads

Filtering statistics of sampled reads.

Number of Reads | Percentages

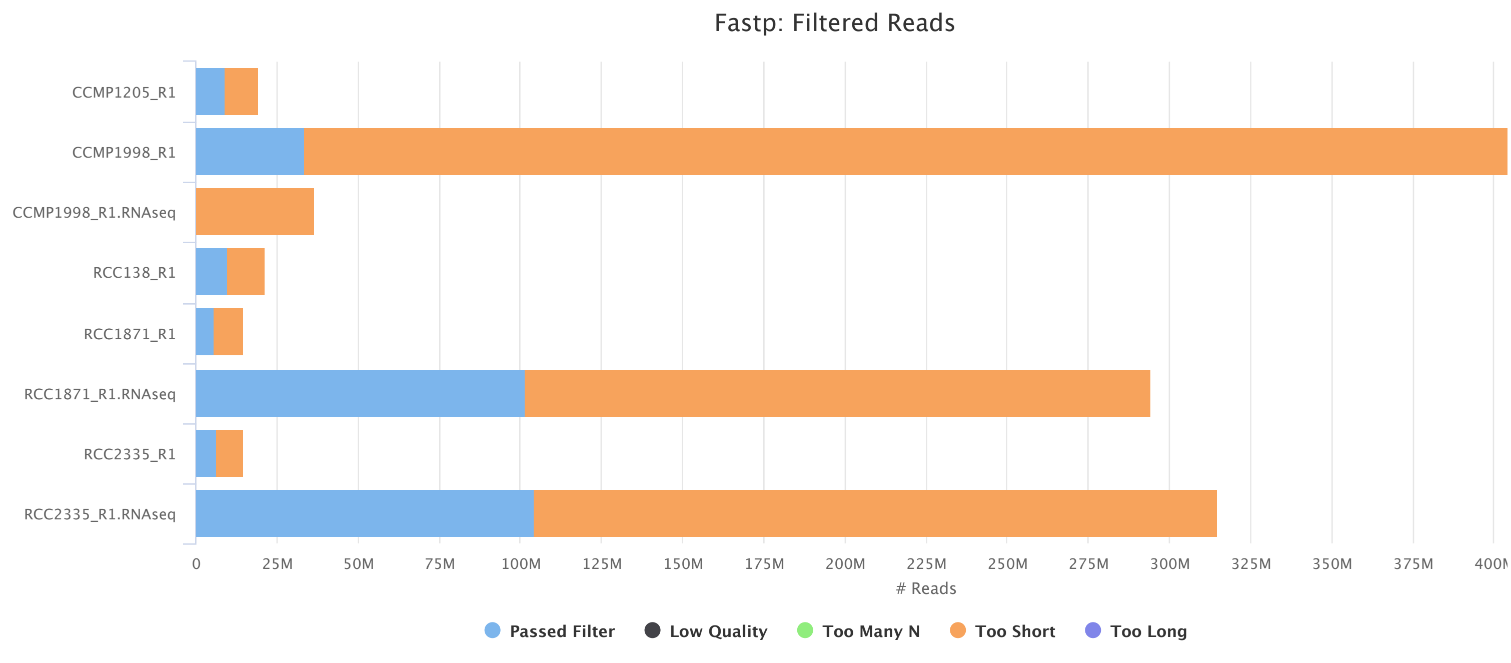

Insert Sizes

Insert size estimation of sampled reads.

Y-Limits: on

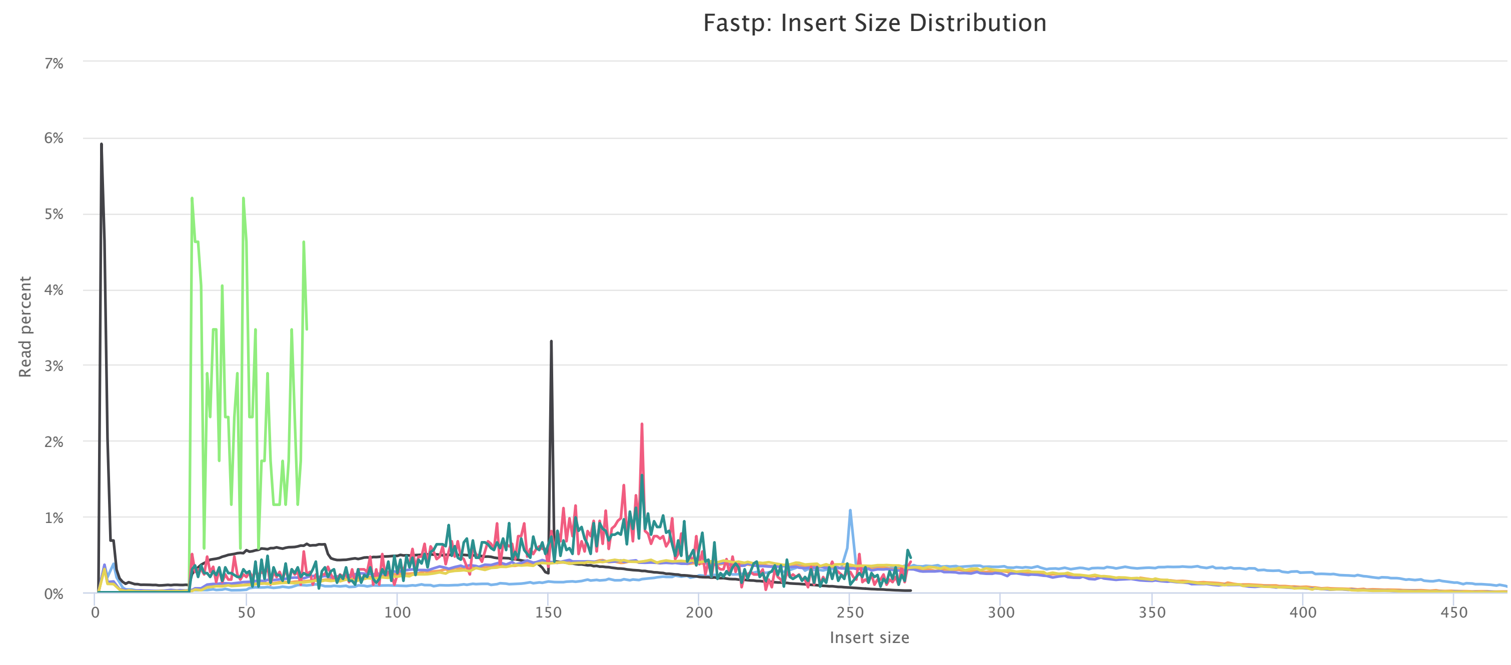

Sequence Quality

Average sequencing quality over each base of all reads.

Y-Limits: on

Read 1: Before filtering | Read 1: After filtering | Read 2: Before filtering | Read 2: After filtering

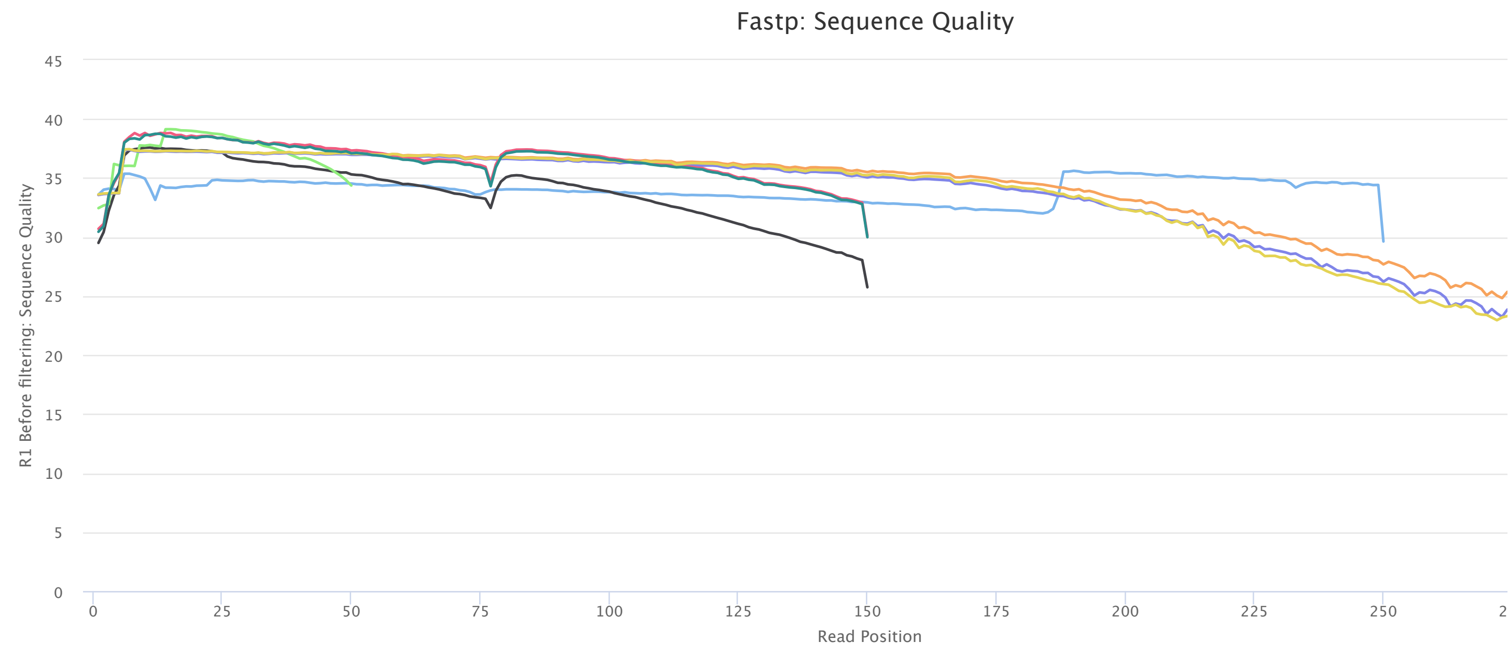

GC Content

Average GC content over each base of all reads.

Y-Limits: on

Read 1: Before filtering | Read 1: After filtering | Read 2: Before filtering | Read 2: After filtering

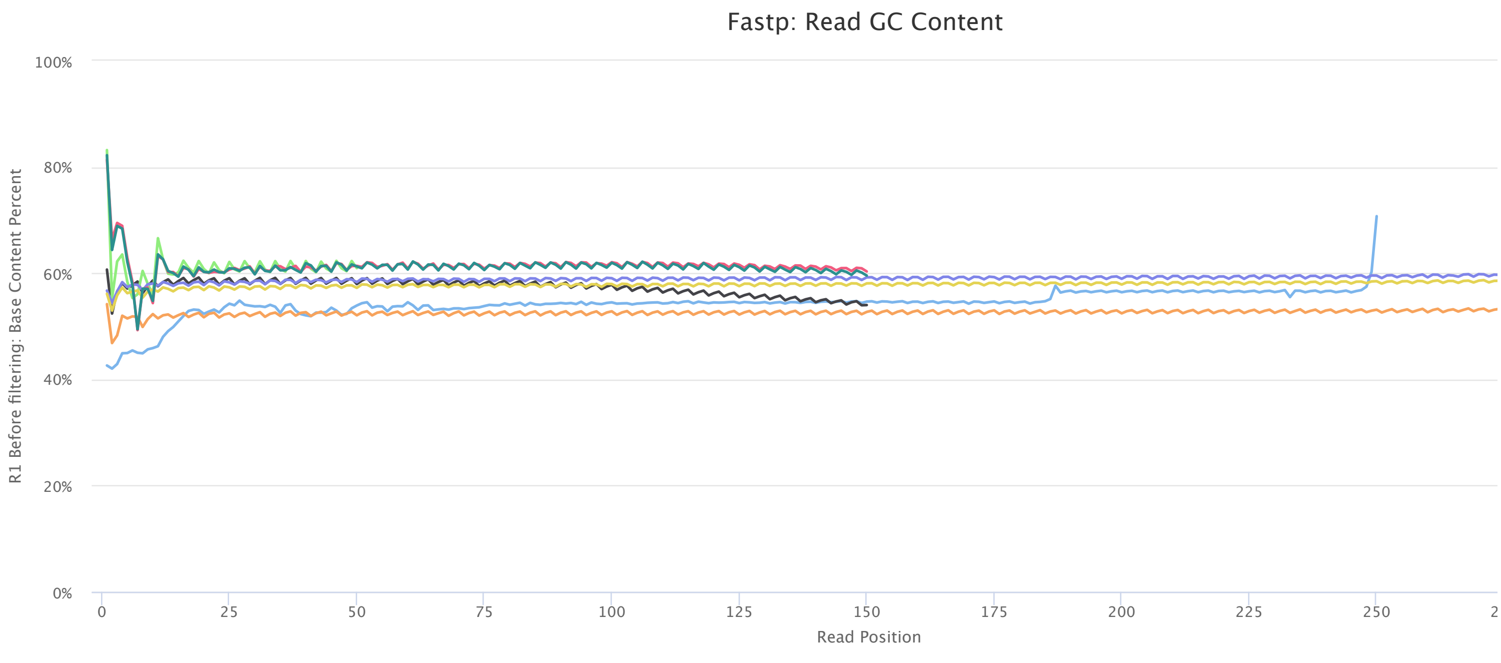

N content

Average N content over each base of all reads.

Y-Limits: on

Read 1: Before filtering | Read 1: After filtering | Read 2: Before filtering | Read 2: After filtering

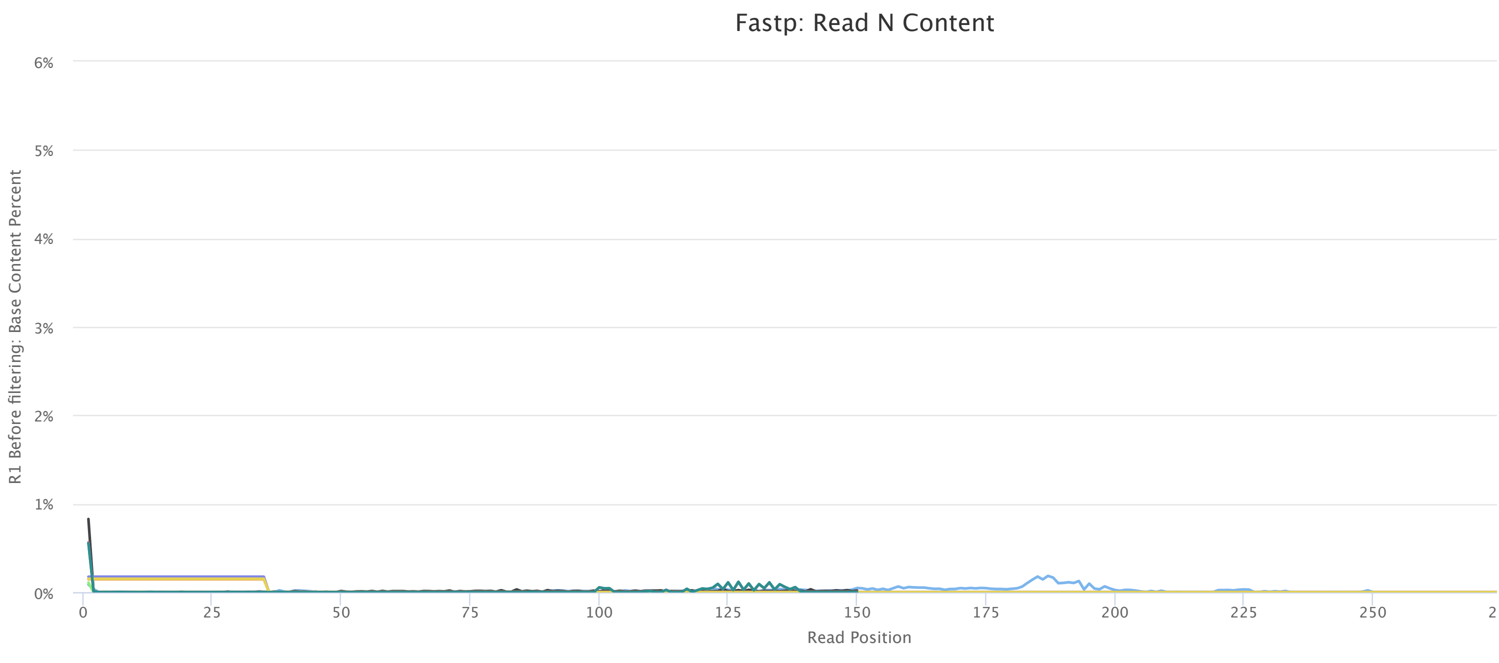

FastQC *Version:* `0.12.1`

FastQC is a quality control tool for high throughput sequence data, written by Simon Andrews at the Babraham Institute in Cambridge.

Sequence Counts

Sequence counts for each sample. Duplicate read counts are an estimate only.

Number of reads | Percentages

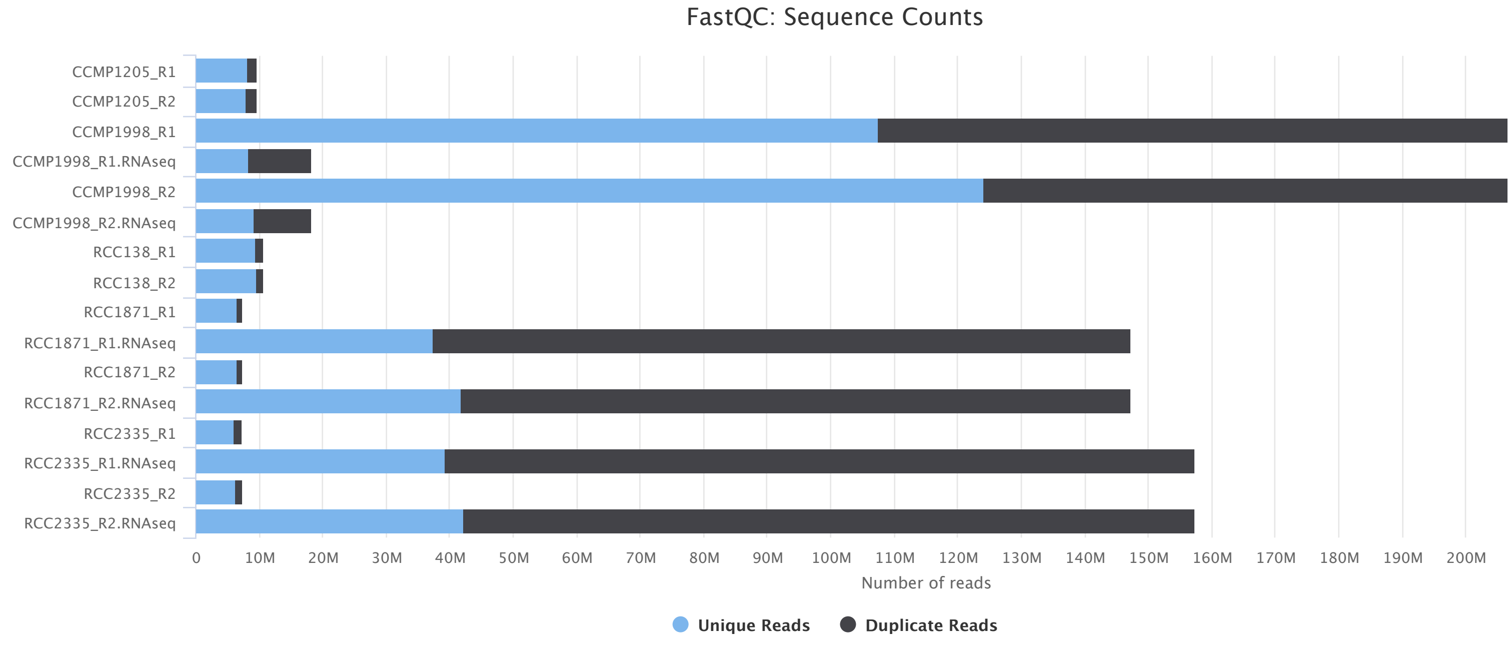

Sequence Quality Histograms 7 3

The mean quality value across each base position in the read.

Y-Limits: on

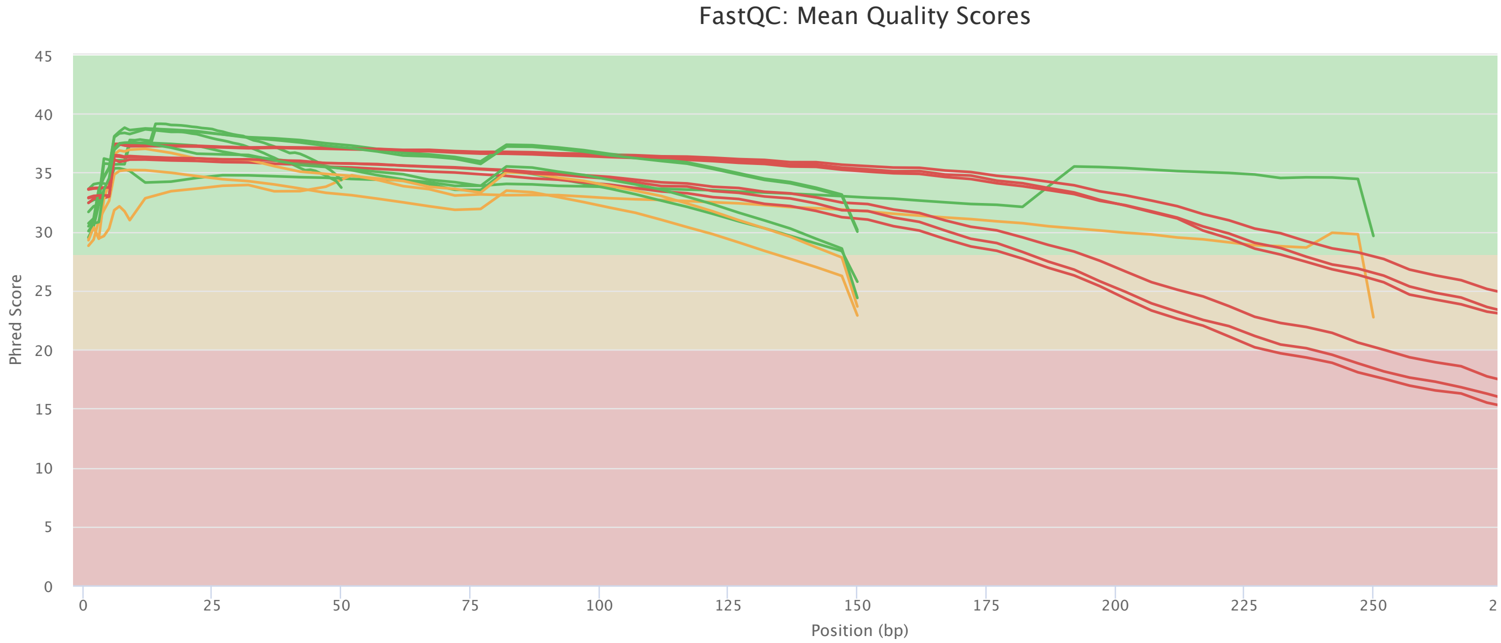

Per Sequence Quality Scores 16

The number of reads with average quality scores. Shows if a subset of reads has poor quality.

Y-Limits: on

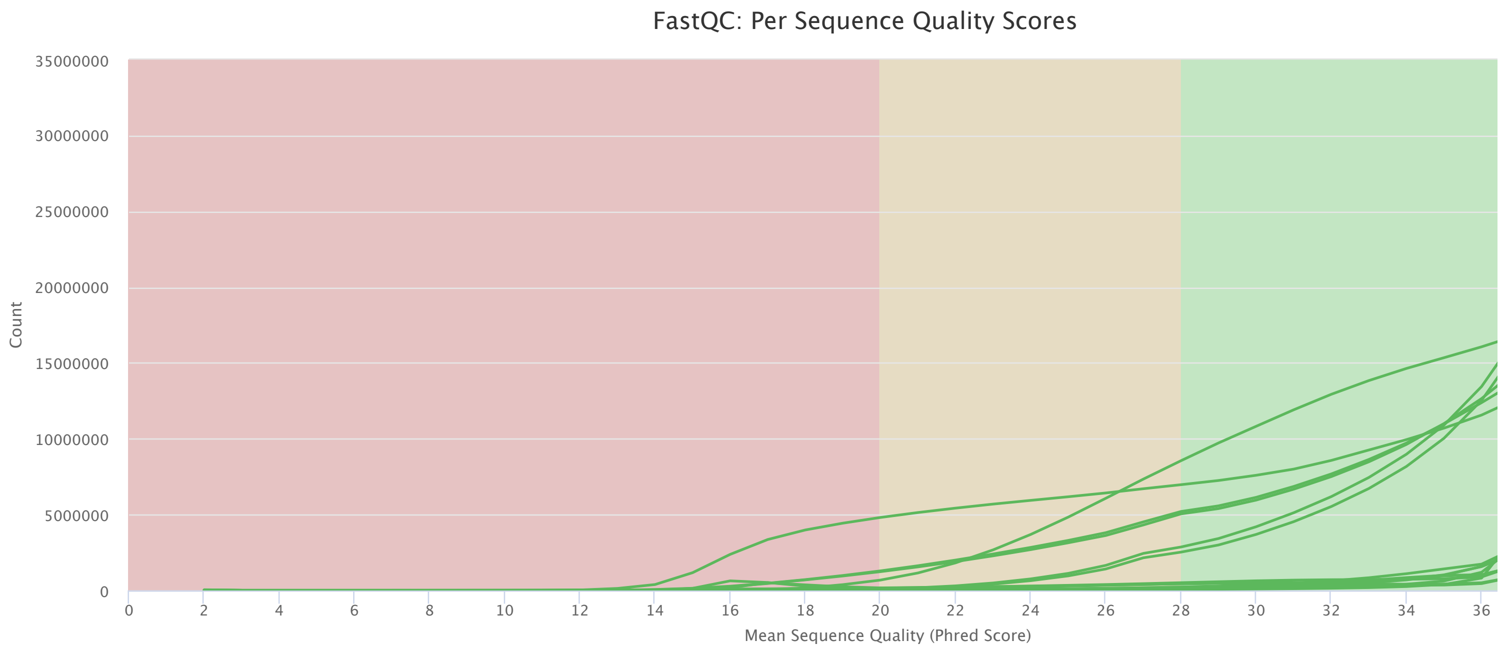

Per Base Sequence Content 6

The proportion of each base position for which each of the four normal DNA bases has been called.

Click a sample row to see a line plot for that dataset.

Rollover for sample name

Position: - %T: - %C: - %A: - %G: -

Export Plot

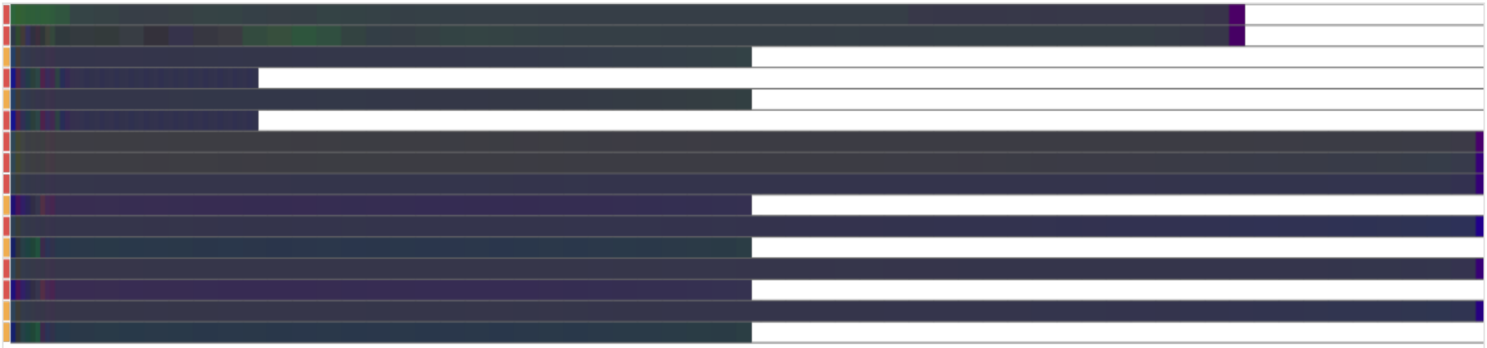

Per Sequence GC Content 9

The average GC content of reads. Normal random library typically have a roughly normal distribution of GC content.

Y-Limits: on

Percentages | Counts

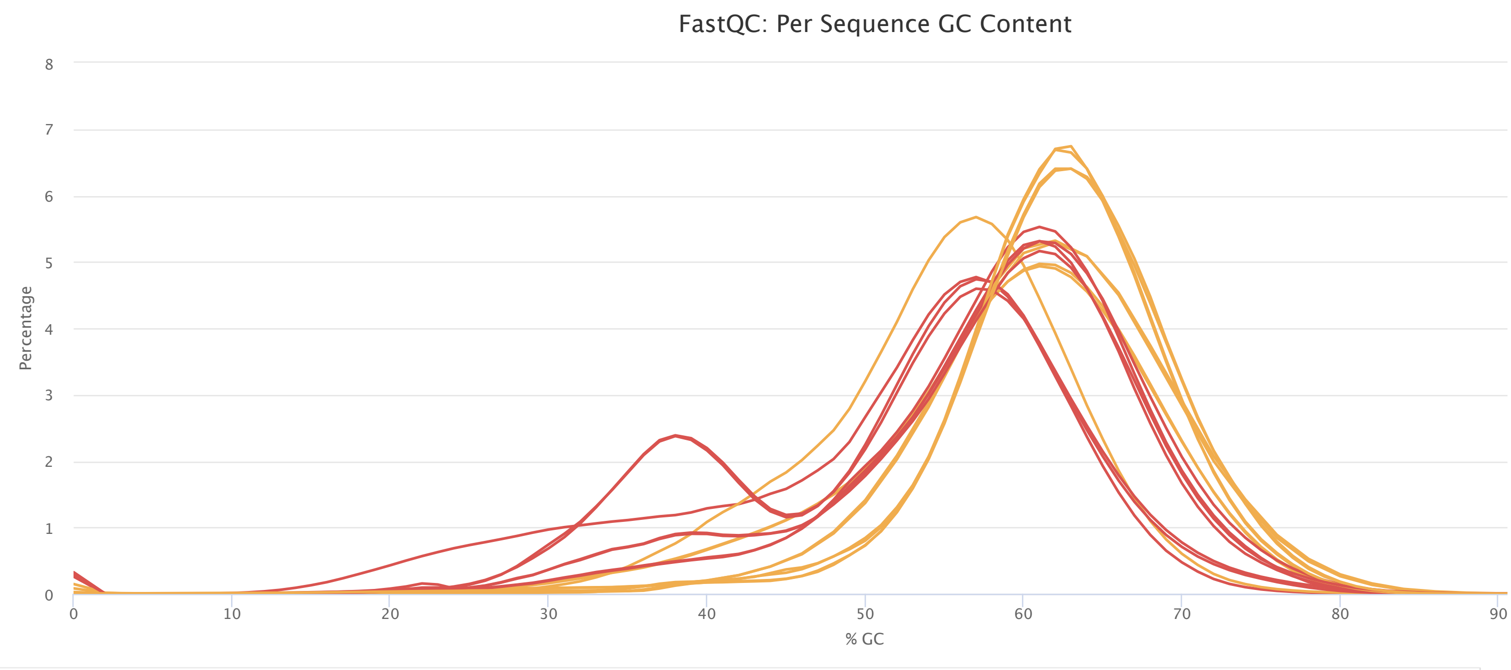

Per Base N Content 16

The percentage of base calls at each position for which an N was called.

Y-Limits: on

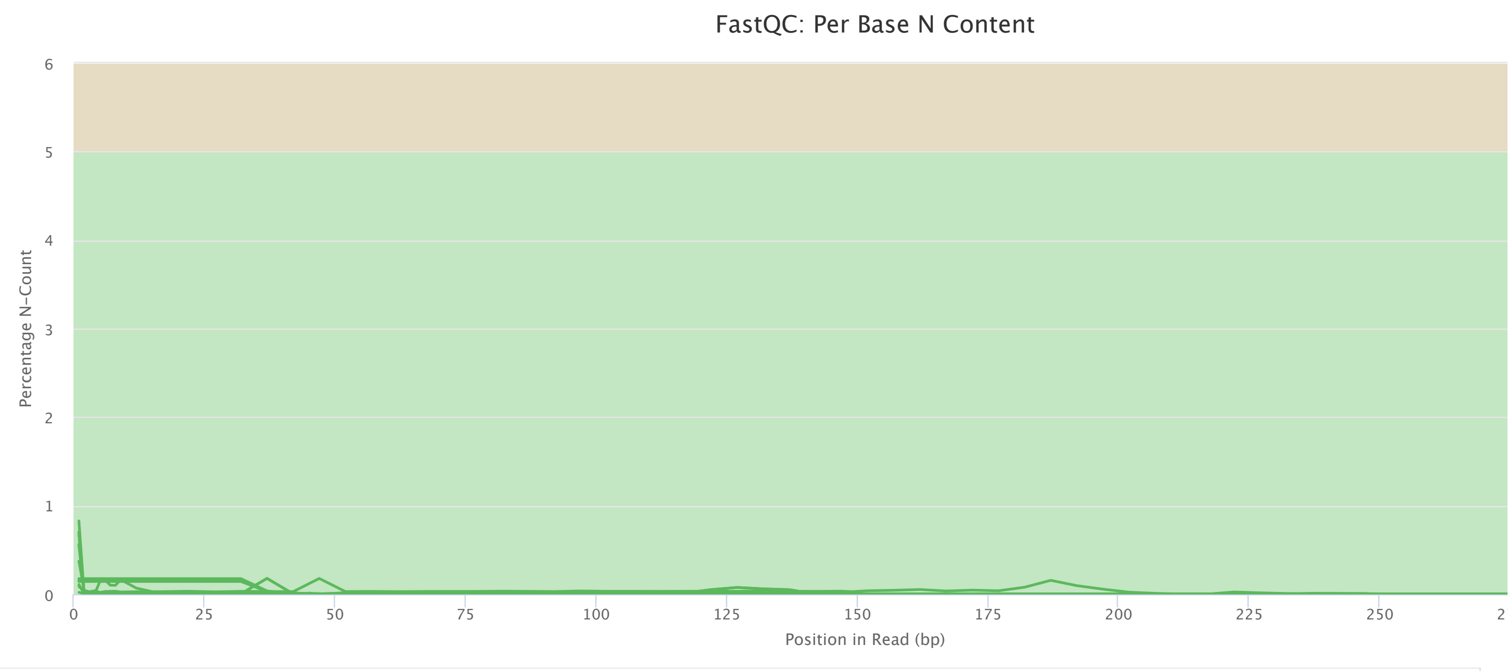

Sequence Length Distribution

8 8

The distribution of fragment sizes (read lengths) found. See the FastQC help

Y-Limits: on

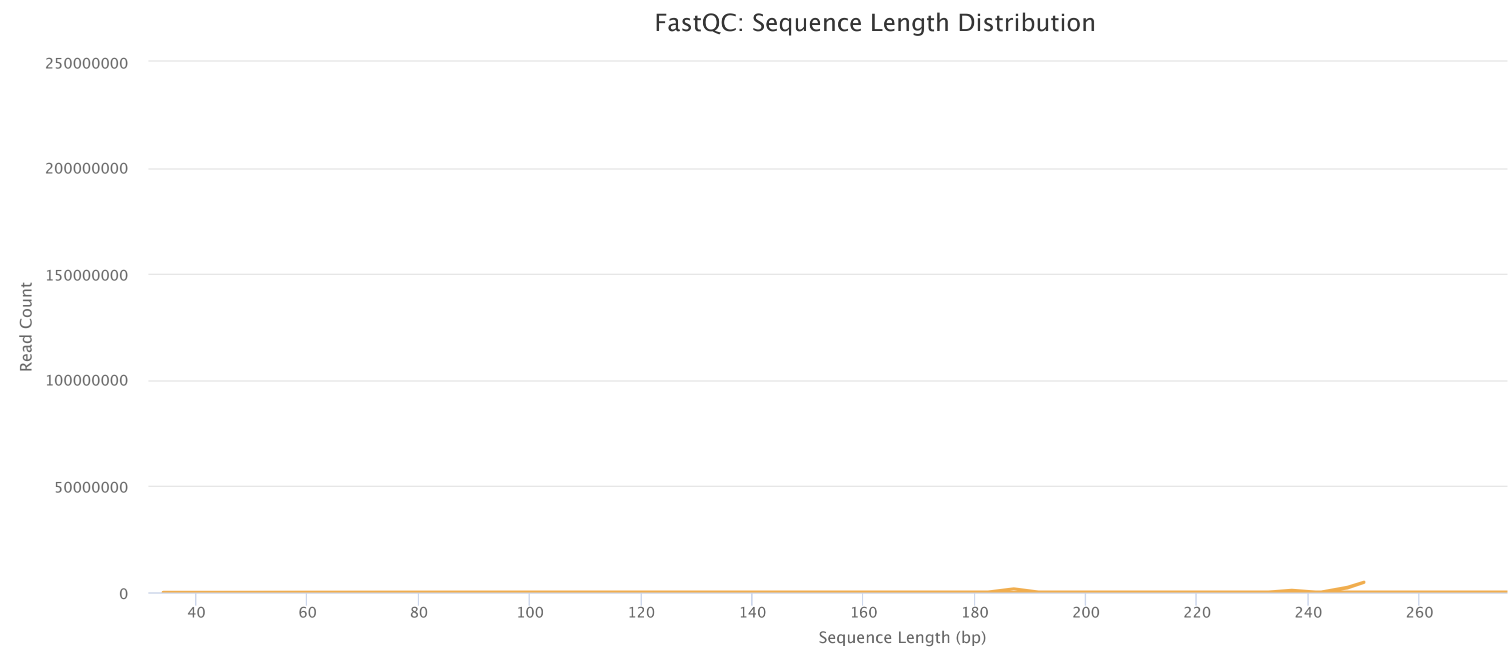

Sequence Duplication Levels

8 3

The relative level of duplication found for every sequence.

Y-Limits: on

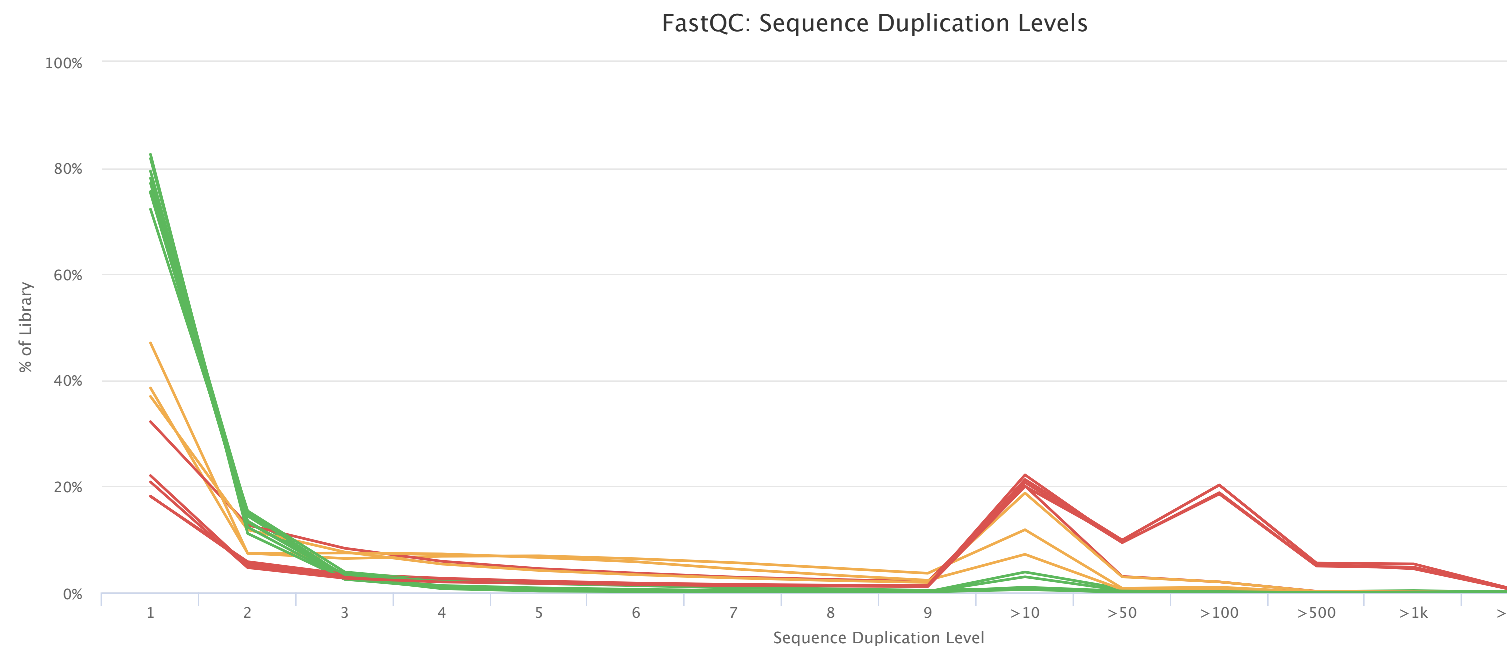

Overrepresented sequences by sample 4 12

The total amount of overrepresented sequences found in each library.

16 samples had less than 1% of reads made up of overrepresented sequences

Top overrepresented sequences

Top overrepresented sequences across all samples. The table shows 20 most overrepresented sequences across all samples, ranked by the number of samples they occur in.

Copy table | Configure Columns | Plot | Showing 9/9 rows and 3/3 columns.

Overrepresented sequence	Samples	Occurrences	% of all reads
NNNNNNNNNNNNNNNNNNNNNNNNNNNNNI	6	78 952	0.0069%
TTTTTTTTTTTTTTTTTTTTTTTTTTTTTTTTTTT TTTTTTT	2	429 793	0.0378%
AAAAAAAAAAAGCACACGTCTGAACTCC ACTAGCTTATCTCGTATG	1	14 958	0.0013%
AAAAAAAAAAACACACGTCTGAACTCCA CTAGCTTATCTCGTATGC	1	10 511	0.0009%
CGTCGTGTAGGGAAAGAGTGTAGATCTC(GTCGCCGTATCATTAAAA	1	60 298	0.0053%
GATCGGAAGAGCACACGTCTGAACTCCA CCGCTCATTATCTCGTAT	1	781 009	0.0687%
GATCGGAAGAGCGTCGTGTAGGGAAAGA AGGATAGGGTGTAGATCT	1	783 050	0.0689%
GATCGGAAGAGCACACGTCTGAACTCCA CGAGATTCCATCTCGTAT	1	756 445	0.0666%
GATCGGAAGAGCGTCGTGTAGGGAAAGA TCAGAGCCGTGTAGATCT	1	764 496	0.0673%

Adapter Content 8 4

The cumulative percentage count of the proportion of your library which has seen each of the adapter sequences at each position.

Y-Limits: on

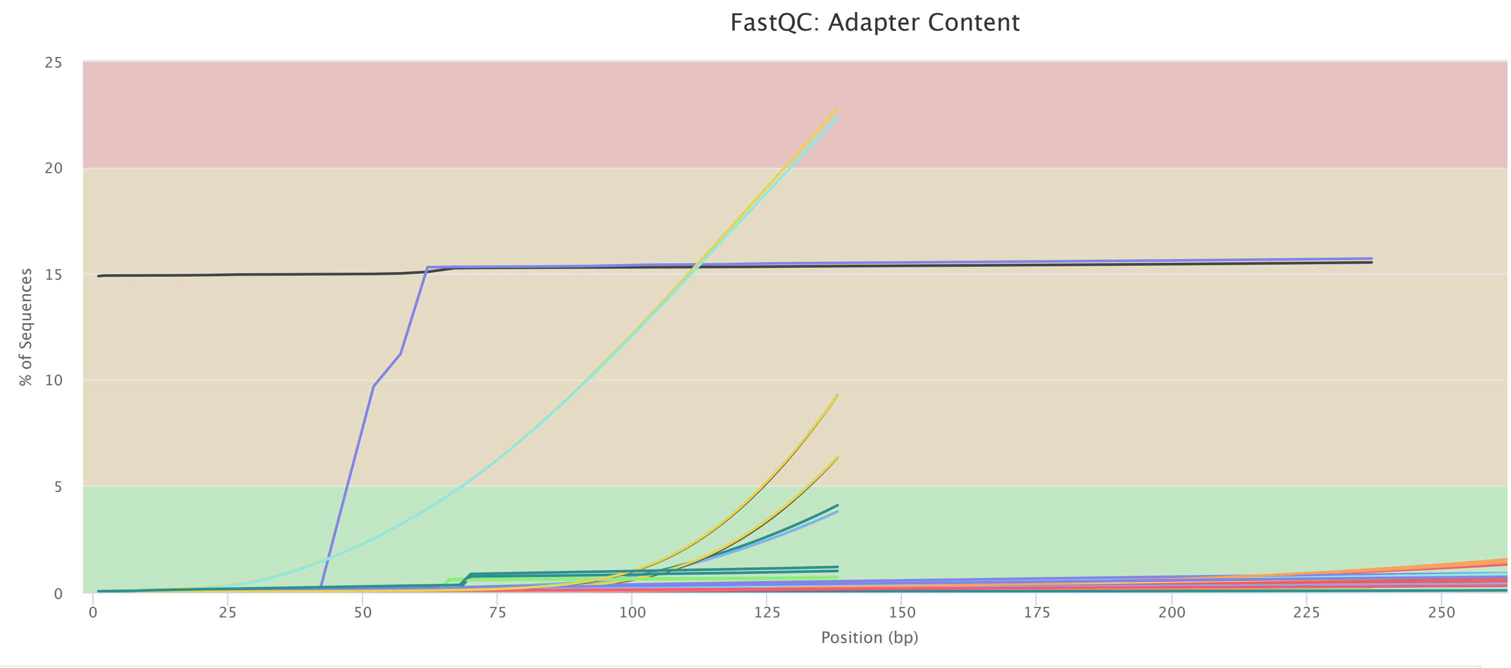

Status Checks

Status for each FastQC section showing whether results seem entirely normal (green), slightly abnormal (orange) or very unusual (red).

Sort by highlight Min: 0 Max: 1

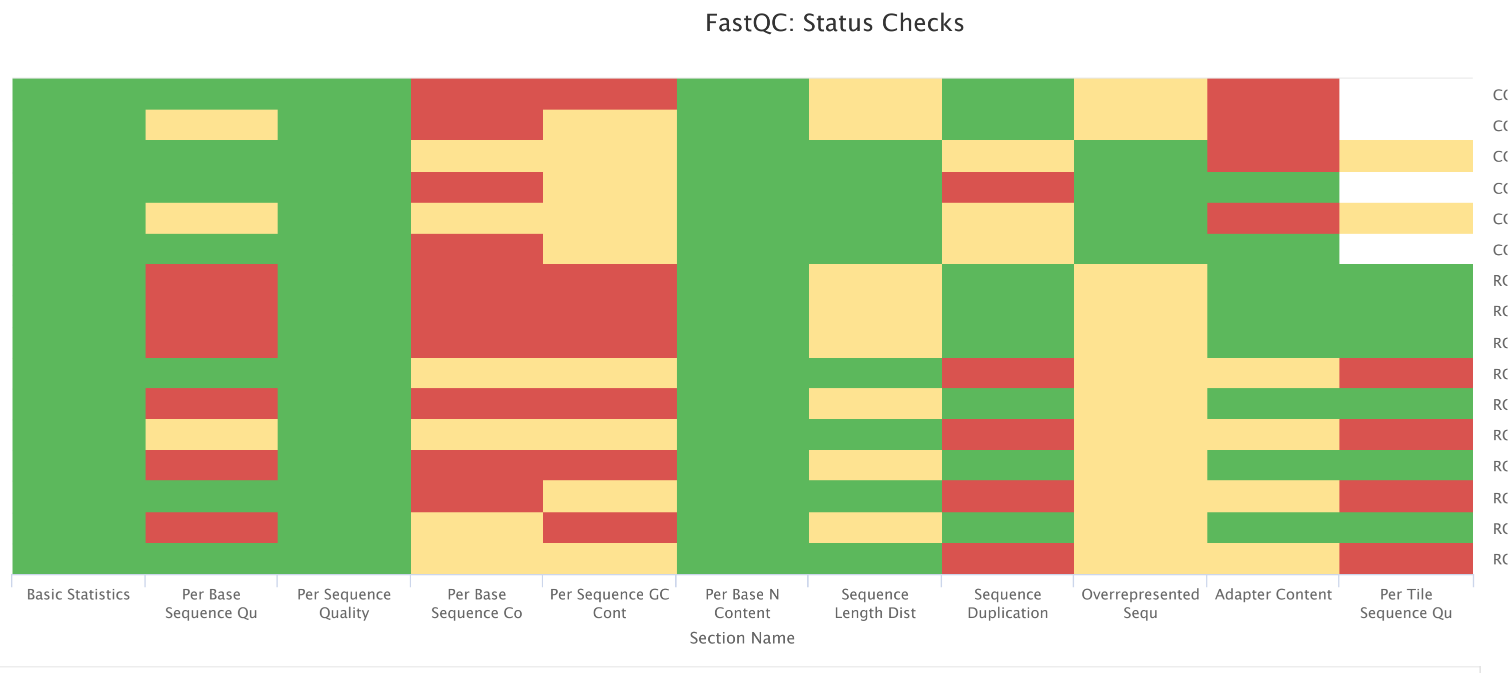

Software Versions

Software Versions lists versions of software tools extracted from file contents.

Copy table

Software	Version
BUSCO	5.6.1
FastQC	0.12.1
fastp	0.23.4

MultiQC v1.19 - Written by Phil Ewels, available on GitHub.
This report uses HighCharts, jQuery, jQuery UI, Bootstrap, FileSaver.js and clipboard.js.
